# Persistent circulation of Coxsackievirus A6 of genotype D3 in mainland of China between 2008 and 2015

**DOI:** 10.1038/s41598-017-05618-0

**Published:** 2017-07-14

**Authors:** Yang Song, Yong Zhang, Tianjiao Ji, Xinrui Gu, Qian Yang, Shuangli Zhu, Wen Xu, Yi Xu, Yong Shi, Xueyong Huang, Qi Li, Hong Deng, Xianjun Wang, Dongmei Yan, Wei Yu, Shuang Wang, Deshan Yu, Wenbo Xu

**Affiliations:** 1 WHO WPRO Regional Polio Reference Laboratory and Ministry of Health Key Laboratory for Medical Virology, National Institute for Viral Disease Control and Prevention, Chinese Center for Disease Control and Prevention, Beijing, People’s Republic of China; 2 Anhui University Of Science and Technology, Anhui Province, People’s Republic of China; 3 Yunnan Center for Disease Control and Prevention, Kunming, Yunnan Province People’s Republic of China; 4 Shaanxi Center for Disease Control and Prevention, Xi’an, Shaanxi Province People’s Republic of China; 5 Jiangxi Center for Disease Control and Prevention, Nanchang, Jiangxi Province People’s Republic of China; 6 Henan Center for Disease Control and Prevention, Zhengzhou, Henan Province People’s Republic of China; 7 Hebei Center for Disease Control and Prevention, Shijiazhuang, Hebei Province People’s Republic of China; 8 Xinjiang Uygur Autonomous Region Center for Disease Control and Prevention, Urumqi City, Xinjiang Uygur Autonomous Region, Ürümqi, People’s Republic of China; 9 Shandong Center for Disease Control and Prevention, Jinan, Shandong Province People’s Republic of China; 10 Liaoning Center for Disease Control and Prevention, Shenyang, Liaoning Province People’s Republic of China; 11 Jilin Center for Disease Control and Prevention, Changchun, Jilin Province People’s Republic of China; 12 Gansu Center for Disease Control and Prevention, Lanzhou, Gansu Province People’s Republic of China

## Abstract

A total of 807 entire *VP1* sequences of Coxsackievirus A6 (CV-A6) from mainland of China from 1992 to 2015, including 520 in this study and 287 from the GenBank database, were analysed to provide a basic framework of molecular epidemiological characteristics of CV-A6 in China. Sixty-five *VP1* sequences including 46 representative CV-A6 isolates from 807 Chinese strains and 19 international strains from GenBank were used for describing the genotypes and sub-genotypes. The results revealed that CV-A6 strains can be categorised into 4 genotypes designated as A, B, C, and D according to previous data and can be further subdivided into B1–B2, C1–C2, and D1–D3 sub-genotypes. D3 is the predominant sub-genotype that circulated in recent years in mainland of China and represents 734 of 807 Chinese isolates. Sixty-six strains belong to D2, whereas B1 and C1 comprise a single strain each, and five AFP strains formed B2. Sub-genotype D3 first circulated in 2008 and has become the predominant sub-genotype since 2009 and then reached a peak in 2013, while D2 was mostly undetectable in the past years. These data revealed different transmission stages of CV-A6 in mainland of China and that sub-genotype D3 may have stronger transmission ability.

## Introduction

Human enterovirus (EV), which belongs to the genus *Enterovirus*, family *Picornaviridae*, is a small non-enveloped, positive sense, single-stranded RNA virus^1^. EV infections are associated with a wide spectrum of illnesses, including mild diseases like a febrile illness and sometimes severe neurological disorders such as aseptic meningitis, myocarditis, acute flaccid paralysis, and encephalitis. Human EVs are classified into 4 species: EV-A, EV-B, EV-C, and EV-D; the EV-A species now consists of 25 serotypes: Coxsackievirus group A (CV-A; serotypes 2–8, 10, 12, 14, and 16); newly identified EVs (serotypes EV-A71, A76, A89–92, A114, A119–121); simian enteroviruses SV19, SV43, and SV46; and baboon enterovirus A13^2–6^.

Hand, foot, and mouth disease (HFMD) is a common epidemic disease that affects young children and causes a series of symptoms such as a fever, sore throat, general rash on the hands and feet, and exanthema in the oral cavity and on the tongue. In rare cases, however, patients may also develop neurological complications such as neurogenic pulmonary oedema, aseptic meningitis, and acute flaccid paralysis; thus, this virus has become a significant public health threat worldwide^7–9^. More than 90% of HFMD cases are caused by EV-A, especially by EV-A71 and CV-A16. EV-A71 is more often associated with neurological diseases and sometimes death as compared to the other EV-A serotypes, whereas CV-A16-associated HFMD is usually mild^10, 11^. Nonetheless, in recent years, CV-A6-associated HFMD outbreaks have taken place worldwide, first in Finland in 2008, and then this disease circulated in France, Spain, and other European countries from 2009 to 2011. CV-A6 was the predominant pathogen for the subsequent HFMD outbreaks in these countries^12–14^. From 2014 to 2015, CV-A6-associated HFMD outbreaks occurred again in France^15^. Outbreaks in North America from 2011 to 2013 have also been reported^16^. In Asia, CV-A6 was responsible for HFMD outbreaks in Singapore in 2009, Japan in 2011, Thailand in 2012, and mainland of China in 2013^17–21^. Since 2013, repeated larger-scale HFMD outbreaks caused by CV-A6 have occurred in China, and CV-A6 has become an important virus on the HFMD pathogen spectrum, even replaced EV-A71 and CV-A16 as the leading HFMD pathogen in many Provinces of China in 2013 and 2015^22, 23^.

The clinical features of CV-A6-associated HFMD are slightly different from those caused by other EVs and represent so-called atypical HFMD, including spread of lesions beyond the typical sites of HFMD: to the trunk, neck, legs, and perioral area, and onychomadesis which causes nail loss^24, 25^. Severe cases caused by CV-A6 were also reported in China, characterised by high fever with shorter duration and twitching, as compared with EV-A71-associated severe cases^26^, and sometimes cause brainstem encephalitis and aseptic meningitis^27^. In addition to the largest number of HFMD cases, CV-A6 causes a large number of severe HFMD cases; therefore, CV-A6-related HFMD has been gradually becoming an important part of HFMD surveillance. Furthermore, CV-A6 is an important pathogen of herpangina^14^, but herpangina is not a disease that needs to be reported in the disease surveillance reporting system of China. Thus, the burden of the disease caused by CV-A6 infection may be strongly underestimated.

The *VP1* coding region contains many important neutralisation epitopes and was demonstrated to help identify EV serotypes^28^. A phylogenetic dendrogram based on the entire *VP1* capsid sequences of EV has been used for discrimination of genotypes; this approach is effective during temporal and geographical analysis of different outbreaks^29^. Studies have proven the correlation between the genotype identity and molecular epidemiological characteristics of EV-A71 and CV-A16^29, 30^. Nonetheless, there has been a lack of systematic research on classification of genotypes of CV-A6 strains based on the entire *VP1* region; therefore, comprehensive application of bioinformatics methods is performed in this study to expound the genetic evolution of CV-A6 circulating in China and to improve the understanding of its genetic characteristics and genotypic and transmission patterns. The results of this study will provide important basic data for the prevention and control of enteroviral diseases in China and has crucial public health significance and practical utility for the development of effective HFMD prevention and control measures.

## Results

### Geographic and annual distribution of Chinese CV-A6 isolates

**Figure 1:**
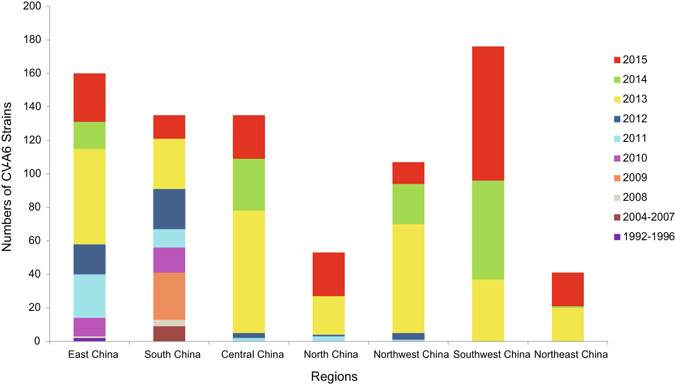
Geographic and annual distribution of 807 Chinese CV-A6 strains included in this study.

A total of 520 Chinese CV-A6 sequences collected in this study and 287 Chinese CV-A6 sequences downloaded from the GenBank database were used here and are summarised in Supplementary Materials (Supplementary Table S1). These 807 strains were isolated from 21 Provinces (or municipalities and autonomous regions), which represent 7 geographic regions of China including East China (Shandong, Jiangsu, Zhejiang, Fujian, and Shanghai), South China (Guangdong), Central China (Hunan, Henan, and Jiangxi), North China (Tianjin, Hebei, and Shanxi), Northwest China (Xinjiang, Shaanxi, and Gansu), Southwest China (Sichuan, Yunnan, Guizhou, and Chongqing), and Northeast China (Liaoning and Jilin), during the period 1992–2015 (Supplementary Table S1, Fig. 1). The strains isolated since 2008 are all HFMD-related strains (except that a Shandong strain 08216/SD/CHN/2008 was isolated from a patient with aseptic meningitis). Only 2 CV-A6 isolates were obtained between 1992 and 1996 and 9 isolates were reported between 2004 and 2007; strains isolated between 2008 and 2012 accounted for 18.8% (152/807) of all isolates, and strains isolated between 2013 and 2015 accounted for 79.8% (644/807; Supplementary Table S2).

### Molecular phylogeny of modern CV-A6

First of all, a database of the CV-A6 entire *VP1* sequences (915 bp) was built. We filtered the entire *VP1* sequences of 65 CV-A6 strains by constructing a phylogenetic tree based on all the CVA-6 sequences released before October 10^th^, 2016, from the GenBank database plus 520 strains in this study, and excluded the laboratory adaptive strains, clones, strains with high passage numbers, and probably incorrect strains, meanwhile consulting other research on CV-A6, to ensure the representativeness of the chosen strains. The database covers CV-A6 strains isolated between 1949 (the prototype Gdula strain) and 2015 from 7 countries including 46 from mainland of China (between 1992 and 2015) (Table 1).

**Table 1:** Isolates used for molecular analysis of CV-A6.

Isolation year	Countries	Strain name	GenBank accession No.	Genotype/Sub-genotype	Origin
1949	USA	Gdula	AY421764	A	GenBank
1992	China	92022/SD/CHN/1992	JQ364886	B1	GenBank
1996	China	96188/SD/CHN/1996	JQ364887	C1	GenBank
2011	Japan	shizuoka 1	AB649286	D3	GenBank
1999	Japan	Kyoto1	AB779614	D1	GenBank
1999	Japan	Hyogo1278	LC126143	D1	GenBank
2004	China	AFP560/GD/CHN/2004	KP143074	B2	GenBank
2005	China	AFP262/GD/CHN/2005	KP143075	B2	GenBank
2006	China	AFP569/GD/CHN/2006	KP143077	D2	GenBank
2007	China	AFP051/GD/CHN/2007	KP143078	B2	GenBank
2008	China	08216/SD/CHN/2008	JQ364888	D2	GenBank
2008	China	JB14080448	KC866900	D2	GenBank
2008	Finland	Finland/2008	KM114057	D3	GenBank
2008	India	N-313 IND	JN203517	C2	GenBank
2008	Spain	ESP08/1023	FR797984	D3	GenBank
2008	Spain	ESP08/54694	FR797987	D3	GenBank
2008	Spain	ESP08/54698	FR797988	D1	GenBank
2009	China	JB143090122	KC866916	D2	GenBank
2009	China	JB143090119	KC866921	D2	GenBank
2009	Japan	Kyoto4	AB779617	D2	GenBank
2009	Taiwan, China	TW/20/2009	JQ946050	D3	GenBank
2010	China	10MF66 Q4 JS	KJ577275	D3	GenBank
2010	China	10032/SD/CHN/2010	JQ364889	D2	GenBank
2010	China	CVA6-SHZH2010-0601	JX154921	D2	GenBank
2010	China	CVA6-SHZH2010-0905	JX154931	D3	GenBank
2010	China	10MF67 JS/CHN/2010	KJ577276	D3	GenBank
2010	China	109097/GZ/CHN/2010/	KJ865424	D3	GenBank
2010	France	CF140007 FRA	HE572906	D3	GenBank
2010	France	CF165026 FRA	HE572917	D1	GenBank
2010	France	CF175076 FRA	HE572928	D3	GenBank
2010	France	CF175080 FRA	HE572930	D1	GenBank
2010	France	CF180026 FRA	HE572932	D1	GenBank
2010	France	CF181032 FRA	HE572935	D1	GenBank
2010	France	CF194028 FRA	HE572938	D3	GenBank
2010	Taiwan, China	TW/409/2010	JQ946055	D3	GenBank
2011	China	HN421/HN/CHN/2011	JN797598	D2	GenBank
2011	China	CVA6-SHZH2011-0507	JX473340	D2	GenBank
2011	China	CVA6-SHZH2011-0701	JX473370	D3	GenBank
2011	China	SHAPHC1883/SH/CHN/2011	JX495130	D3	GenBank
2011	China	11MC31 Q2	KJ577283	D3	GenBank
2011	China	2011FJLY035/FJ/CHN/2011	KJ743208	D3	GenBank
2011	China	075/GS/CHN/2011	KY211690	D2	this study
2011	China	FH058/SX/CHN/2011	KY211724	D2	this study
2012	China	SHAPHC4703/SH/CHN/2012	KC481614	D3	GenBank
2012	China	54203/HeB/CHN/2012	KY211711	D2	this study
2013	China	TJ13-54-C/CHN/2013	KJ848308	D3	GenBank
2013	China	A023/YN/CHN/2013	KY424358	D2	this study
2013	China	13-9/HeN/CHN/2013	KY424393	D2	this study
2013	China	13-110/HeN/CHN/2013	KY424419	D2	this study
2013	China	13-97/JL/CHN/2013	KY424390	D2	this study
2013	China	13-107/JL/CHN/2013	KY424420	D3	this study
2013	China	54106/HeB/CHN/2013	KY424359	D3	this study
2013	China	13-87/SaX/CHN/2013	KY424394	D3	this study
2013	China	13-08/SC/CHN/2013	KY424424	D3	this study
2013	China	13-28/ZJ/CHN/2013	KY424409	D3	this study
2014	China	AYLA14083/HN/CHN/2014	KU708581	D3	GenBank
2014	China	J044/YN/CHN/2014	KY211729	D3	this study
2014	China	14-106/HuN/CHN/2014	KY424386	D3	this study
2014	China	14-26/SaX/CHN/2014	KY424381	D3	this study
2014	China	14-106/JX/CHN/2014	KY424385	D3	this study
2015	China	SHAPHC5696/SH/CHN/2015	KU736939	D3	GenBank
2015	China	A120/YN/CHN/2015	KY424357	D3	this study
2015	China	15-43/JX/CHN/2015	KY424371	D3	this study
2015	China	DY033/SD/CHN/2015	KY211721	D3	this study
2015	China	18-62/LN/CHN/2015	KY424360	D3	this study

**Figure 2:**
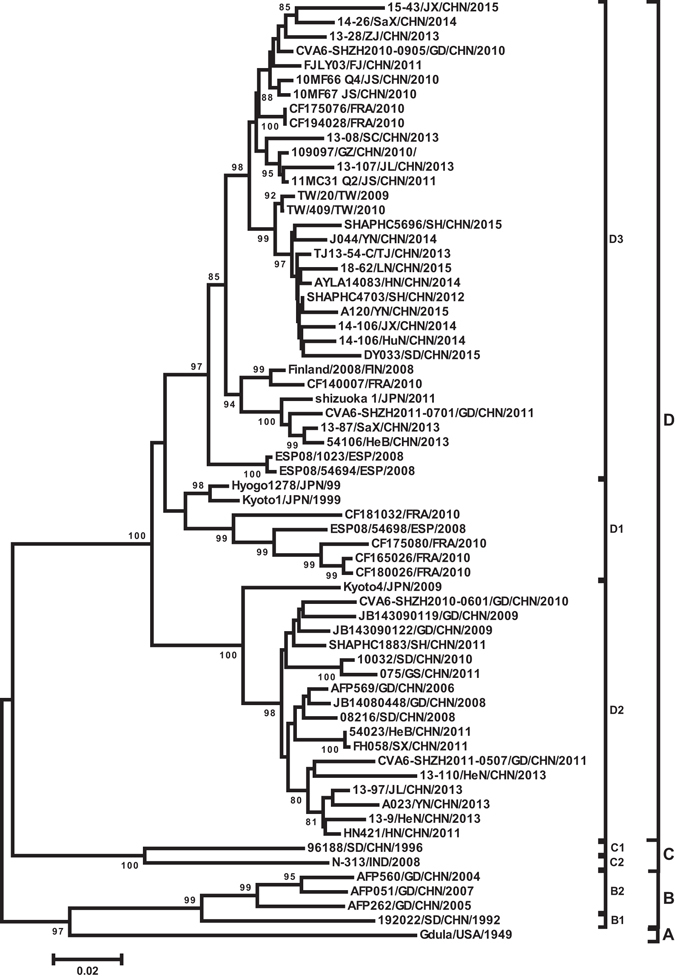
Phylogenetic trees based on the complete VP1 nucleotide sequences of CV-A6 of 65 representative strains isolated between 1949 (the prototype Gdula strain) and 2015. Numbers at the nodes indicate bootstrap support for each node (percentage of 1000 bootstrap replicates). A difference of at least 15% in the entire *VP1* region of CV-A6 strains was used to distinguish genotypes. CV-A6 strains are labelled using the following format: ‘isolate name’/‘country of origin’/‘year of isolation’ (details are listed in Table 1). Different genotypes and sub-genotypes are shown to the right of the tree and the scale at the bottom indicates measurement of relative phylogenetic distance. Abbreviations of countries/Province: CHN, China; JPN, Japan; IND, India; TW, Taiwan, China; FIN, Finland; FRA, France; ESP, Spain.

A phylogenetic dendrogram was constructed with the 65 CV-A6 *VP1* sequences using the neighbour-joining method in MEGA 5.0 (Fig. 2). The bootstrap test was performed with 1000 replications. According to the rule that a difference of at least 15% in the entire *VP1* nucleotide sequences should be used for distinguishing genotypes^30^, the *VP1* tree clearly indicated that all the CV-A6 strains are segregated into 4 distinct genotypes which were designated as A, B, C, and D, referring to the other studies^21, 22^. The Gdula prototype strain isolated in the United States in 1949 formed a single lineage and differed from other stains by 19.1–21.2% and can be classified as the sole member of genotype A. Genotype B consists of 4 strains isolated in mainland of China between 1992 and 2007. Genotype C, represented by 2 strains, includes a virus from Shandong province of China in 1996 and India in 2008.

Genotype D is represented by the rest of 58 strains isolated from 1999 to 2015 in mainland of China, Japan, Finland, Spain, and France. The group mean distances among the 4 genotypes range from 17.7% to 21.2%.

Genotypes B, C, and D can be further subdivided into 2, 2, and 3 sub-genotypes, respectively, with a difference of more than 8% in the *VP1* region (Fig. 2). Cluster B1 contains a single strain isolated from Shandong Province, China, in 1992, and Cluster B2 consists of 3 strains isolated from patients with acute flaccid paralysis (AFP) from Guangdong Province, China, from 2004 to 2007; the mean distance between B1 and B2 sub-genotypes is 9.4%. Furthermore, the 2 strains of genotype C represent 2 sub-genotypes (C1 and C2) independently with a 10.1% *VP1* sequence divergence. As to genotype D, Cluster D1 was composed of strains isolated in Japan in 1999 and in France and Spain from 2008 to 2010. Cluster D2 was composed of strains isolated in Japan and mainland of China from 2006 to 2011. Cluster D3 was composed of strains isolated in Finland, Spain, France, Japan, and China from 2008 to 2015. Isolates within sub-genotype D1 differed from one another by 0.4–6.8% and from strains in sub-genotype D2 and D3 by 8.3–11.7% and 4.1–10.9%, respectively. Viruses in sub-genotype D2 differ from one another by 1.1–6.4% and from strains in sub-genotype D3 by 6.4–12.0%, whereas strains in sub-genotype D3 differ from one another by 0.0–6.7%.

Except for the prototype Gdula strain (genotype A) and Indian strain N-313 (sub-genotype C2), all the other international CV-A6 strains belong to genotype D. On the basis of the above analysis, the outbreaks of CV-A6 that happened in Europe from 2008 to 2010 can be attributed to 2 major sub-genotypes, D1 and D3; however, sub-genotype D3 was responsible for most of the outbreaks worldwide after 2010. These data suggested that the sub-genotype D3 viruses probably have stronger transmissibility, infectivity, and virulence and may be the main reason for the persistent international circulation of CV-A6.

### The pattern of circulation of CV-A6 in mainland of China: the switch from the co-circulation of sub-genotypes D2 and D3 to the predominance of D3

**Figure 3:**
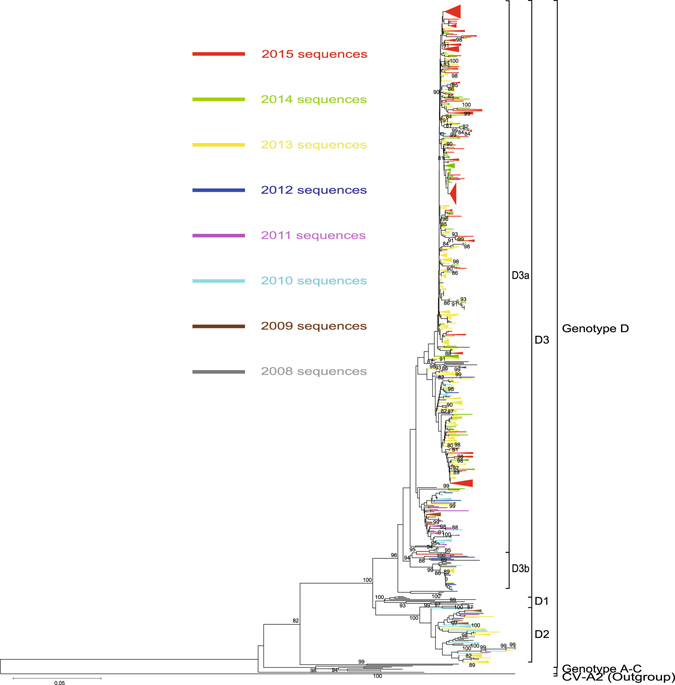
Phylogenetic analyses based on 807 entire *VP1* sequences of CV-A6 in China. The phylogenetic tree shows the evolutionary tendency of sub-genotype D2 and D3 strains in China since 2008; sub-genotype D3 was the predominant one. The branches of sequences from mainland of China are highlighted in different colours according to years from 2008 to 2015, the other international strains (including Taiwan strains) and strains detected before 2008 are uncoloured. The phylogenetic tree indicates that evolutionary branch D3a was responsible for the recent outbreaks since 2013. A ladder-like topology revealed that adaptation of CV-A6 occurred during the epidemic history. A prototype CV-A 2 strain (Fleetwood) served as an outgroup.

Phylogenetic trees were constructed using 807 Chinese entire CV-A6 *VP1* sequences plus the selected 53 international CV-A6 strains, and the prototype CV-A2 strain (Fleetwood) served as an outgroup (Fig. 3).

According to our previous research, all the CV-A6 strains isolated since 2008 circulating in mainland of China clustered with isolates of sub-genotypes D2 and D3, and most of them belong to D3^21^. Strains 92022/SD/CHN/1992 and 96188/SD/CHN/1996 were the sole members of sub-genotypes B1 and C1, respectively. Five Guangdong AFP isolates (strains AFP024/GD/CHN/2004, AFP560/GD/CHN/2004, AFP262/GD/CHN/2005, AFP265/GD/CHN/2005, and AFP051/GD/CHN/2007) formed sub-genotype B2. Furthermore, sub-genotype D1 is composed of the strains free from Chinese sequences; therefore, it can be concluded that the molecular epidemiology of CV-A6 in China since 2008 reflects the pattern of endemic circulation of sub-genotype D2 and D3 viruses.

Sub-genotype D2 (66/807) consists mostly of the strains isolated from 2008 to 2011 and clustered with the Japanese strains isolated during the same period. This observation indicated that Chinese sub-genotype D2 strains co-evolved and co-circulated with those from neighbouring countries. Most of the sub-genotype D2 strains isolated between 2008 and 2011 were from South and East China especially from Guangdong Province, where they gave rise to the first reported CV-A6-associated HFMD circulation in mainland China^31, 32^. Nevertheless, most sub-genotype D2 strains isolated after 2011 were isolated outside South China, suggesting that the transmission route of sub-genotype D2 viruses in China was from south to north. In addition, 4 AFP strains (AFP569, AFP101, AFP147, and AFP149) isolated before 2008 clustered with D2, and originated from Guangdong Province as well. The above finding indicates that D2 genotype in China was probably the first circulating genotype in Guangdong for years, and then triggered the outbreaks in some southern cities, and then was transmitted nationally. The isolates from the European outbreaks did not appear in D2; this result further showed that D2 originated in Asian countries.

The sequences of D3 were analysed further. It was shown that 90.1% (734/807) of the Chinese isolates belong to sub-genotype D3 (except that 1 Guangdong strain was isolated in October 2008, and all the strains have been isolated since 2009), which revealed that sub-genotype D3 was the predominant sub-genotype circulating in mainland of China (Fig. 3b). Most of the Guangdong strains from the CV-A6 emergence period can be seen in this sub-genotype; this observation indicated that during the pre-outbreak period 2009–2012, D2 and D3 co-circulated, but D3 was the major sub-genotype.

Further research split sub-genotype D3 CV-A6 into 2 evolutionary branches, which were designated as D3a and D3b, respectively, the mean distance between these 2 branches reached 6.0% (Fig. 3). The stains that belonged to evolutionary branch D3a (700/734) have been isolated since 2009 (only 1 exception, from 2008), and evolutionary branch D3b (34/734) consists of the Chinese strains from 2011 to 2013 (with only 1 exception: a 2015 Yunnan CV-A6 strain, which independently formed a single lineage, suggesting that it might have been imported from another country) and clustered with the Finnish strains in 2008, the French strains in 2010, and the Japanese strains between 2010 to 2013. As we can see from evolutionary branch D3a of D3, most of the Chinese strains belong to this cluster, and almost all the strains detected since 2014 can be found only in D3a (except for a Yunnan CV-A6 strain in D3b and 2 Xinjiang strains in D2). This finding suggested that the D3a branch may be responsible for the recent virus circulation and may have high epidemic activity. Therefore, we can hypothesise that most descendants from evolutionary branch D3a were responsible mainly for the current large CV-A6-related HFMD outbreaks.

### Annual and geographic distribution of D2 and D3 genotypes (D3a and D3b branches) circulating in mainland of China since 2008

**Figure 4:**
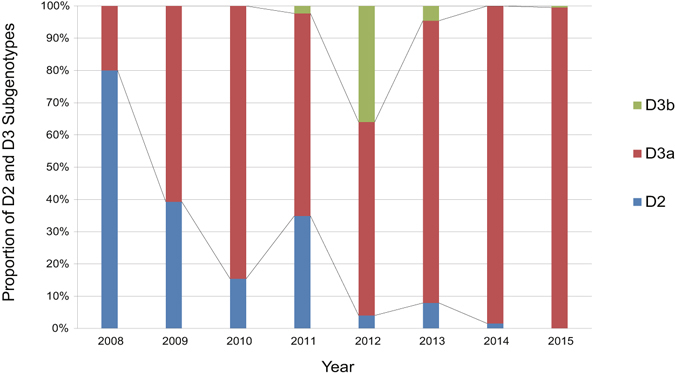
Yearly proportional distribution of HFMD-associated sub-genotypes D2 and D3 (evolutionary branches D3a and D3b) from 2008 to 2015 in mainland of China.

In 2008, 80% (4/5) CV-A6 isolates from this period belonged to sub-genotype D2, 4 were isolated in Guandong and 1 in Shandong Province (Fig. 4). Because 4 Guangdong AFP isolates, which were detected before 2008, also belonged to sub-genotype D2, we can speculate that D2 may be the predominant sub-genotype of CV-A6 circulation in mainland of China before 2009. Nonetheless, since 2009, sub-genotype D2 viruses have been gradually decreasing in prevalence and a large number of strains from D3 have been detected. From 2009 to 2014, the percentages of isolates from sub-genotype D2 were 39.3% (11/28), 15.4% (4/25), 34.9% (15/43), 4.0% (2/50), 7.9% (24/305), and 1.5% (2/131), respectively, and no strains were detected in 2015. As to sub-genotype D3, strains from evolutionary branch D3b can be found only in 2011, 2012, and 2013 (except for 1 Yunnan strain from 2015), which suggests that evolutionary branch D3b circulated within a limited period. On the contrary, since 2009, strains from D3a have started to boom; in the CV-A6 outbreak year 2013, 87.5% (267/305) CV-A6 strains belonged to evolutionary branch D3a, 98.5% (129/131) in 2014, and 99.5% (207/208) in 2015. These data indicate that viruses from D3a were responsible for the recent CV-A6 outbreaks.

**Figure 5:**
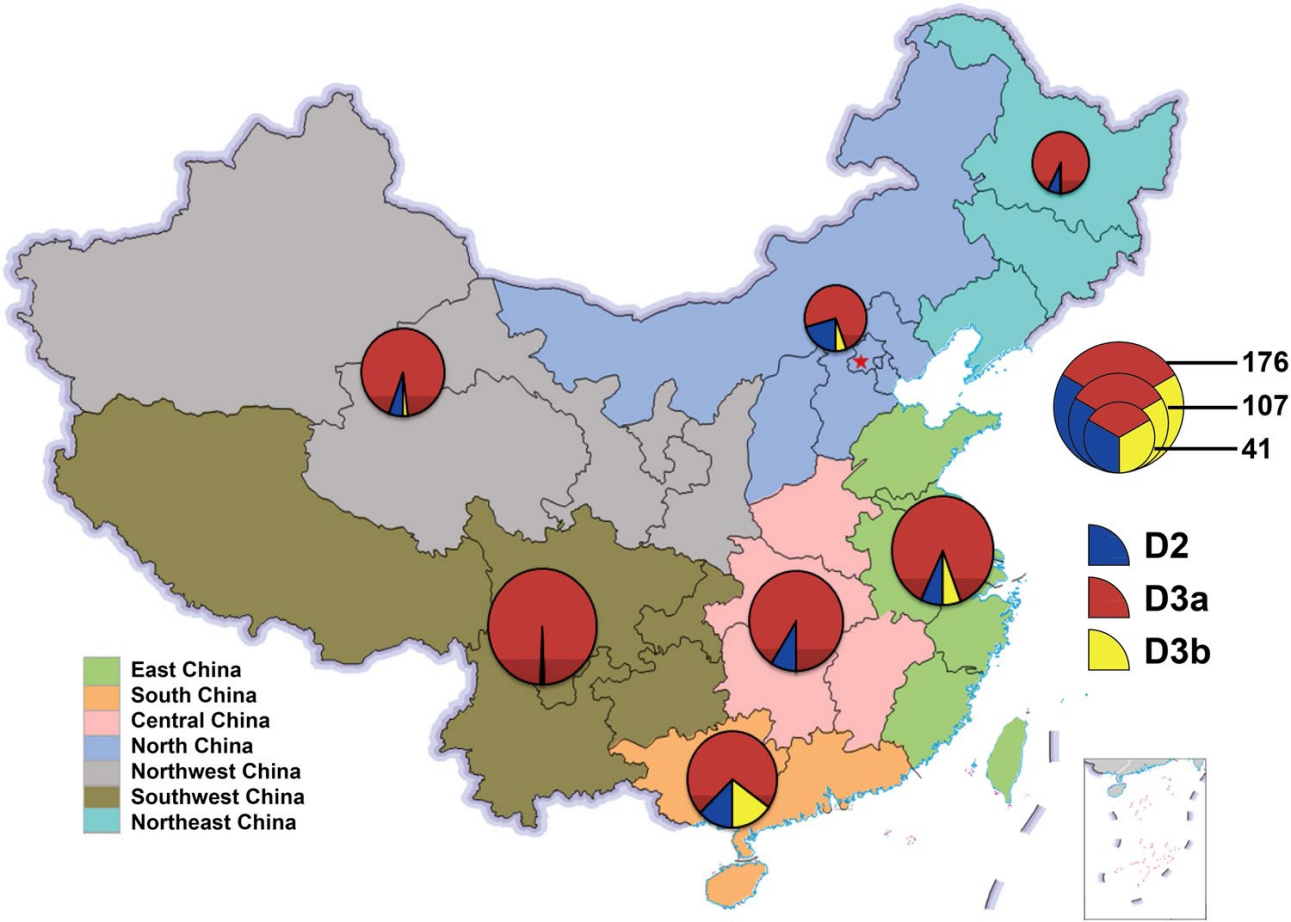
The geographic distribution of HFMD-associated CV-A6 strains in 7 representative regions of China, the region and the number of the sequences are coloured according to the legend (Taiwan sequences ﻿are not included). The free map source was acquired from d-maps.com (URL: http://d-maps.com/carte.php?num_car=15272&lang=en; the content is properly attributed in the appropriate figure legend according to the attribution guidelines; the website is protected by Copyright France, Registration Number: 58KU297). The map was then generated and modified using Microsoft Excel 2010 software.

For the geographic distribution of HFMD-related sub-genotypes that circulated in mainland of China, the D3a evolutionary branch from the D3 sub-genotype has been predominant in all 7 regions of China since 2008 (Fig. 5). Sub-genotype D2 can be also seen in all 7 regions but only constituted a small proportion. This finding indicated again that since 2008, D2 and D3 sub-genotypes have co-circulated in mainland of China but D3, especially evolutionary branch D3a, has been the major one. As to evolutionary branch D3b, the strains from this branch can be detected only in 4 regions: East China, South China, North China, and Northwest China, and mostly (82.4%, 28/34) in Southern and East China, suggesting that D3b might be responsible for the small-scale transmission and probably was imported from the neighbouring countries.

## Discussion

The HFMD epidemics have become a serious public health threat in the Asia-Pacific region especially in China in the past decade^33, 34^. Systematic surveillance of HFMD established in mainland of China in 2008 has been contributing a lot to HFMD research. Nonetheless, studies have focused mostly on the predominant serotypes EV-A71 and CV-A16, whereas information about other EVs is still limited because other EV types usually have only caused sporadic cases. In 2013 and 2015, a series of CV-A6-associated HFMD outbreaks were reported in many provinces of China, and CV-A6 even surpassed EV-A71 and CV-A16 to become the predominant pathogen^21–23^. Since then, more and more researchers have given attention to the CV-A6 serotype. The reasons for the repeated larger-scale HFMD outbreaks and a large number of severe cases caused by CV-A6 remain unclear.

Although molecular epidemiologic analyses of Chinese CV-A6 have been conducted in limited geographic areas and periods^21, 22^, those studies may have some limitations. Firstly, previous research always have more or less following problems on the division of genotypes and sub-genotypes: (1) The division of genotypes was based on only partial *VP1* sequences of CV-A6; (2) The division of genotypes was not following the consensus principle (a difference of at least 15% in the entire *VP1* nucleotide sequences should be used for distinguishing genotypes, 8% for sub-genotypes), but according to the shape of the clusters in the phylogenetic dengrogram, which was too subjective. Secondly, currently, there has no published works of Chinese CV-A6 based on a relatively complete time span which includes the CV-A6 from the two large-scale outbreaks (2013 and 2015). Thirdly, because of the vast territory of China, the terrain and climate are different in different regions, which may lead to diverse spread and duplicate abilities of CV-A6. Therefore, an overall research based on the nationwide CV-A6 sequences is very important. The previous evolutionary analysis of CV-A6 of small-scale outbreaks may be quite different from national-based outbreaks. Therefore, a comprehensive study of CV-A6 molecular epidemiology covering most of Chinese areas, on a long-term timescale, and a phylogenetic analysis based on adequate entire *VP1* nucleotide sequences of CV-A6 was still lacking. A unified classification of CV-A6 genotypes and sub-genotypes was still unclear.

However, in this study, we have precisely and comprehensively segregated the genotypes and sub-genotypes of worldwide CV-A6 based on the entire *VP1* nucleotide sequences (915 bp), and then roughly revealed the circulation pattern of CV-A6 worldwide. Moreover, we accomplished a comprehensive molecular epidemiological analysis of CV-A6 from all seven regions in mainland of China, and analysed based on a long time span of 2008–2015, trying to find the explanations for the recent outbreaks.

The phylogenetic analysis indicated that the CV-A6 strains were segregated into 4 genotypes (A, B, C, and D). Still, like EV-A71 and CV-A16^30, 35^, the prototype strain of CV-A6 (strain Gdula/USA/1949) is the sole member of genotype A, and genotypes B, C, and D can be further subdivided into sub-genotypes B1–B2, C1–C2, and D1–D3, respectively. Most of the international strains cluster with sub-genotypes D3, suggesting that sub-genotype D3 may be responsible most of the circulation of CV-A6 worldwide.

According to our phylogenetic analysis of Chinese CV-A6 based on the entire *VP1* sequences, the sub-genotype switch within genotype D occurred in the past decade. The Chinese CV-A6 strains that were associated with HFMD in the past 10 years are all clustered within genotype D. The most recent descendants of sub-genotype D3 viruses (especially evolutionary branch D3a) are the majority of CV-A6 strains detected during the HFMD outbreaks in China since 2013. In addition, the strains isolated from HFMD outbreaks in 2014 to 2015 can only be seen in evolutionary branch D3a (except for a Yunnan strain and 2 Xinjiang strains). These findings suggest that the evolution of sub-genotype D3 CV-A6 viruses is responsible for the CV-A6-related HFMD, and particularly the evolutionary branch D3a CV-A6 has caused large-scale outbreaks since 2013. The concept of evolutionary branch D3a has never been put forward in the previous studies, and it may have important public health significance in China.

To gain a better understanding of the molecular epidemiology of CV-A6 in mainland of China, the circulation of CV-A6 in China could be divided into three evolutionary stages based on our results above. The first stage should be called the sporadic stage that occurred before 2009, in which sub-genotype D2 might be the predominant sub-genotype of CV-A6. From 2009 to 2012, CV-A6 started to circulate from Guangdong Province and then gradually became one of the primary HFMD pathogens in different regions in China: this is the second stage, which could be regarded as the pre-large-scale outbreak stage. The isolates from this phase, which underwent sub-genotype transition co-circulated in both sub-genotype D2 and D3, and mostly in the D3a evolutionary branch. Evolutionary branch D3b was probably responsible for the small-scale transmission in Shenzhen and Shanghai city. At the third stage (since 2013, we can call it the large-scale outbreak stage), many outbreaks have occurred one after another in China, and the vast majority of the strains detected during this outbreak stage belong to the D3a branch of sub-genotype D3. In addition, there were very few isolates detected at this stage belonging to sub-genotype D2; after 2013, the strains that did not belong to branch D3a were nearly undetectable (only 1 Yunnan strain belonged to D3b and 2 Xinjiang strains belonged to D2). In a word, the decreasing prevalence of strains from the D2 genotype combined with the booming viruses from the D3 sub-genotype summarise the circulation pattern of CV-A6 in mainland of China.

In general, after the emergence and continued evolution, whether a genotype (or sub-genotype) will persistently circulate for a relatively long period like EV-A71 and CV-A16 did^29, 33, 36^, and whether genotype D3 CV-A6 will still remain the predominant pathogen that causes HFMD in the future in China, continuous surveillance for the coming years should be enhanced.

Large-scale HFMD outbreaks caused by CV-A6 that occurred in China suggest that CV-A6 has been becoming more aggressive in terms of virulence and transmissibility. Nevertheless, the relation between clinical features and molecular characteristics of CV-A6 is still unclear. Other studies have indicated that recombination events that happened in the *2C* region of CV-A6 might be relevant to the more generalised rash^22, 37^; however, considerable studies need to be conducted to demonstrate the molecular evidence, which is our urgent and crucial point for the further research on CV-A6. The 520 CV-A6 entire VP1 sequences from 18 provinces (or municipalities and autonomous regions) involved in this study were obtained through HFMD case routine surveillance in mainland of China, which greatly enriched the GenBank database.

For prevention and control of HFMD, the vaccine strategy may be the most effective way, and should protect the population from infective viruses. Recently, more and more studies on EV-A71 vaccines modified them stepwise, and EV-A71 vaccines went through a clinical trial and have been put to use in mainland of China since 2016^38–40^. Nonetheless, because CV-A6 is emerging as the predominant causative pathogen of HFMD with a huge number of severe cases in mainland of China, there is an urgent need to develop an effective vaccine against CV-A6 infection. Several studies on the CV-A6 vaccine based on a mouse model have yielded some results^41, 42^, but further experiments and development are required.

The National Notifiable Disease Reporting System (NNDRS) of HFMD organized in 2008 in mainland of China has been requiring the specific serotypes information of HFMD cases for EV-A71 and CV-A16, but not for CV-A6. Therefore, the molecular epidemiology of EV-A71 and CV-A16 nationwide has been maturely studied, but the molecular epidemiology of CV-A6 nationwide has been finitely focused on. However, in recent years, CV-A6 has become an important virus in the HFMD pathogen spectrum in China, research vacancies of Chinese CV-A6 nationwide may have a negative impact on China’s public health. Therefore, being the only laboratory in mainland of China that owns CV-A6 isolates from all regions of the nation, this may be the first study or even the only study in recent years that carried out a systematic research on the molecular epidemiology of CV-A6 in mainland of China.

In conclusion, the comparatively thorough molecular epidemiological study of Chinese CV-A6 indicates that CV-A6 has experienced a sub-genotype switch from the co-circulation of sub-genotypes D2 and D3 to the predominance of sub-genotype D3 along with the CV-A6-associated HFMD outbreaks in 2013. Most descendants of sub-genotype D3 are mostly CV-A6 strains isolated during the outbreaks since 2013. The growing virulence and transmissibility and increasing numbers of severe HFMD cases of CV-A6 infection call for further research to overcome the inherent problems of CV-A6.

## Methods

### Ethics Statement

This study did not involve human participants or human experimentation, the only human materials used were stool samples, throat swab samples, and vesicles collected from HFMD patients for public health purposes at the urging of the Ministry of Health, P. R. of China, and written informed consent for the use of their clinical samples was obtained from the parents of the children whose samples were analysed. This study was approved by the second session of the Ethics Review Committee of the National Institute for Viral Disease Control and Prevention (NIVDC), Chinese Center for Disease Control and Prevention, all experimental protocols were approved by NIVDC, and the methods were carried out in accordance with the approved guidelines.

### Virus isolation

The CV-A6 strains used in this study were isolated between 2011 and 2015 from stool, throat swabs, or vesicles from HFMD patients from Yunnan, Zhejiang, Gansu, Guangdong, Guizhou, Hebei, Henan, Hunan, Jiangxi, Jilin, Liaoning, Shaanxi, Shandong, Shanxi, Tianjin, Chongqing, Xinjiang and Sichuan provinces (or municipalities and autonomous regions), which represent different geographic regions of China. Clinical samples were processed according to standard protocols^43^, and were first confirmed as CV-A6 positive by a commercial real-time PCR assay (Shuoshi Biotech, Jiangsu, China). A total of 520 CV-A6-positive samples were selected according to different years and geographic locations. The representative CV-A6-positive samples were inoculated into human rhabdomyosarcoma (RD) and human laryngeal epidermoid carcinoma (HEp-2) cell lines for virus propagation and purification. These 2 cell lines were obtained from the WHO Global Poliovirus Specialized Laboratory, USA, and were originally purchased from the American Type Culture Collection.

### Determination of the entire *VP1* nucleotide sequence of CV-A6

Viral RNA was extracted using a QIAamp Viral RNA Mini Kit (Qiagen, Valencia, CA, USA). Reverse transcription polymerase chain reaction (RT-PCR) was performed to amplify the entire *VP1* capsid region (915 nucleotides) using PrimeScript One Step RT-PCR Kit Ver. 2 (TaKaRa, Dalian, China) with the primers designed in this study (CVA6-VP1-F: 5′-CTTCGTAGTGCCACCAGATA-3′ and CVA6-VP1-R: 5′-GTGGCGAGATGTCGGTTTA-3′). The PCR products were purified using the QIAquick PCR Purification Kit (Qiagen, Germany), and then amplicons were bi-directionally sequenced using ABI 3130 Genetic Analyser (Applied Biosystems, USA)^44^.

### Phylogenetic analysis and genotyping

The entire *VP1* sequence of CV-A6 from the GenBank database was first filtered to exclude the laboratory adaptive strains, clones, strains with high passage numbers, and the possible incorrect strains, and we eventually retrieved 340 entire CV-A6 *VP1* sequences from the GenBank database before October 10th, 2016. In combination with the 520 entire CV-A6 *VP1* nucleotide sequences determined in this study, a total number of 860 strains formed the dataset used in this study, containing 65 strains used for differentiating genotypes (including 46 Chinese strains and 19 international strains) (Table 1), plus other 36 international strains and other 747 Chinese strains (807 Chinese strains and 53 international strains in total) (Supplementary Table S1).

Sequence alignment was conducted by means of the ClustalW tool in MEGA 5.0, the bootstrap test was performed with 1,000 replications. Phylogenetic dendrograms based on complete VP1 coding sequence were constructed by the neighbour-joining method using the Kimura 2-parameter model^45^. Bootstrap values greater than 80% were considered statistically significant for grouping. Genotypes were described in a way similar to that originally used to describe EV-A71 and CV-A16: by the group mean distance computing method in MEGA^45^. A difference of at least 15% in the *VP1* region was used to distinguish genotypes^30^.

### Nucleotide sequence accession numbers

The entire *VP1* nucleotide sequences of the representative CV-A6 strains which represent each sub-genotype and cluster from different years and regions included in this study were deposited in the GenBank database under the accession numbers KY211689–KY211741 and KY424356–KY424426.

## Electronic supplementary material

Supplementary Information
