# Supplementary material for: Persistent circulation of Coxsackievirus A6 of genotype D3 in mainland of China between 2008 and 2015
Source: Sci Rep. 2017 Jul 14;7:5491. doi: 10.1038/s41598-017-05618-0 (PMC5511160; doi:10.1038/s41598-017-05618-0)
Supplement: Supplementary file 1 — Supplementary Information [file 41598_2017_5618_MOESM1_ESM.pdf]

**Persistent circulation of Coxsackievirus A6 of genotype D3 in mainland of China between 2008 and 2015**

Yang Song[1], Yong Zhang[1]*, Tianjiao Ji[1], Xinrui Gu[1], Qian Yang[1], Shuangli Zhu[1], Wen Xu[3], Yi Xu[4], Yong Shi[5], Xueyong Huang[6], Qi Li[7], Hong Deng[8], Xianjun Wang[9], Dongmei Yan[1], Wei Yu[10], Shuang Wang[11], Deshan Yu[12] and Wenbo Xu[1,2]*

**Supplementary Table S1. The districts and time distribution of the CV-A6 VP1 sequences involved in this study.** Numbers of strains collected and sequenced in this study were shaded in grey, those downloaded from GenBank were not shaded

| Regions | Provinces | Numbers of sequences (From this study/GenBank) | | | | | | | | | | | | | | | | | | |
|---|---|---|---|---|---|---|---|---|---|---|---|---|---|---|---|---|---|---|---|---|
| | | 1992~1996 | 2004~2007 | 2008 | 2009 | 2010 | 2011 | | 2012 | | 2013 | | 2014 | | 2015 | | Total | | | |
| East China | Shandong | 2 | | 1 | | 1 | | | | | 6 | | 1 | | 17 | | 24 | 4 | 39 | 121 |
| | Jiangsu | | | | | 7 | | 10 | | 5 | | 10 | | | | | | 32 | | |
| | Zhejiang | | | | | | | | | | 15 | 7 | | | | | 15 | 7 | | |
| | Fujian | | | | | | | 6 | | 6 | | 1 | | | | | | 13 | | |
| | Shanghai | | | | | 3 | | 10 | | 7 | | 18 | | 15 | | 12 | | 65 | | |
| South China | Guangdong | | 9 | 4 | 28 | 15 | | 11 | | 24 | | 30 | | | 14 | | 14 | 121 | 14 | 121 |
| Central China | Hunan | | | | | | | | | | | | 8 | | 4 | | 12 | | 98 | 37 |
| | Henan | | | | | | | 2 | | 3 | 42 | 4 | | 12 | | 16 | 42 | 37 | | |
| | Jiangxi | | | | | | | | | | 27 | | 11 | | 6 | | 44 | | | |
| North China | Tianjin | | | | | | | | | | | 8 | | | 3 | | 3 | 8 | 45 | 8 |
| | Hebei | | | | | | 1 | | 1 | | 11 | | | | 23 | | 36 | | | |
| | Shanxi | | | | | | 2 | | | | 4 | | | | | | 6 | | | |
| Northwest China | Xinjiang | | | | | | | | 4 | | 11 | | 20 | | 1 | | 36 | | 107 | |
| | Shaanxi | | | | | | | | | | 53 | | 2 | | | | 55 | | | |
| | Gansu | | | | | | 1 | | | | 1 | | 2 | | 12 | | 16 | | | |
| Southwest China | Sichuan | | | | | | | | | | 5 | | 2 | | 4 | | 11 | | 176 | |
| | Yunnan | | | | | | | | | | 12 | | 57 | | 73 | | 142 | | | |
| | Guizhou | | | | | | | | | | 17 | | | | | | 17 | | | |
| | Chongqing | | | | | | | | | | 3 | | | | 3 | | 6 | | | |
| Northeast China | Liaoning | | | | | | | | | | 1 | | 1 | | 20 | | 22 | | 41 | |
| | Jilin | | | | | | | | | | 19 | | | | | | 19 | | | |
| Total | | 2 | 9 | 5 | 28 | 26 | 4 | 39 | 5 | 45 | 227 | 78 | 104 | 27 | 180 | 28 | 520 | 287 | 520 | 287 |

**Supplementary Table S2. The 807 CV-A6 strains isolated in mainland of China and 53 international strains (including Taiwan strains) used in molecular epidemiology analysis.**

| Isolated Year | Provinces/ Countries | Strain name | GenBank accession No. | Genotype/ Subgenotype | Origin |
|---|---|---|---|---|---|
| **1949** | USA | Gdula | AY421764 | A | GenBank |
| **1992** | Shandong | 92022/SD/CHN/1992 | JQ364886 | B1 | GenBank |
| **1996** | Shandong | 96188/SD/CHN/1996 | JQ364887 | C1 | GenBank |
| **1999** | Japan | Hyogo1278 | LC126143 | D1 | GenBank |
| **1999** | Japan | Hyogo1283 | LC126144 | D1 | GenBank |
| **1999** | Japan | Kyoto1 | AB779614 | D1 | GenBank |
| **2003** | Japan | Kyoto2 | AB779616 | D1 | GenBank |
| **2004** | Guangdong | AFP024/GD/CHN/2004 | KP143073 | B2 | GenBank |
| **2004** | Guangdong | AFP560/GD/CHN/2004 | KP143074 | B2 | GenBank |
| **2005** | Guangdong | AFP262/GD/CHN/2005 | KP143075 | B2 | GenBank |
| **2005** | Guangdong | AFP265/GD/CHN/2005 | KP143076 | B2 | GenBank |
| **2006** | Guangdong | AFP569/GD/CHN/2006 | KP143077 | D2 | GenBank |
| **2007** | Guangdong | AFP051/GD/CHN/2007 | KP143078 | B2 | GenBank |
| **2007** | Guangdong | AFP101/GD/CHN/2007 | KP143079 | D2 | GenBank |
| **2007** | Guangdong | AFP147/GD/CHN/2007 | KP143080 | D2 | GenBank |
| **2007** | Guangdong | AFP149/GD/CHN/2007 | KP143081 | D2 | GenBank |
| **2007** | Japan | Hyogo3818 | LC126147 | D2 | GenBank |
| **2007** | Taiwan | TW-2007-00141 | KR706309 | D1 | GenBank |
| **2008** | Finland | Fin/Se8717 | KP144344 | D3b | GenBank |
| **2008** | Finland | Fin/Se8841 | KP144345 | D3b | GenBank |
| **2008** | Finland | Fin/Se8926 | KP144346 | D3b | GenBank |
| **2008** | Finland | Finland/2008 | KM114057 | D3b | GenBank |
| **2008** | Guangdong | 2008-EV-390 | KM079502 | D2 | GenBank |
| **2008** | Guangdong | 2008-EV-392 | KM079503 | D2 | GenBank |
| **2008** | Guangdong | JB14080448/2008 | KC866900 | D2 | GenBank |
| **2008** | Guangdong | JB14080583/2008 | KC866901 | D3a | GenBank |
| **2008** | India | N-313 IND | JN203517 | C2 | GenBank |
| **2008** | Shandong | 08216/SD/CHN/2008 | JQ364888 | D2 | GenBank |
| **2008** | Spain | ESP08/54698 | FR797988 | D1 | GenBank |
| **2008** | Spain | ESP08/1023 | FR797984 | D3 | GenBank |
| **2008** | Spain | ESP08/1031 | FR797985 | D3 | GenBank |
| **2008** | Spain | ESP08/1215 | FR797986 | D3 | GenBank |
| **2008** | Spain | ESP08/54694 | FR797987 | D3 | GenBank |
| **2009** | Japan | Hyogo4604 | LC126149 | D2 | GenBank |
| **2009** | Japan | Hyogo4658 | LC126150 | D3a | GenBank |
| **2009** | Japan | Hyogo4667 | LC126151 | D3a | GenBank |
| **2009** | Guangdong | 2009-EV-406 | KM079512 | D2 | GenBank |
| **2009** | Guangdong | 2009-EV-407 | KM079513 | D2 | GenBank |

| | | | | | |
|---|---|---|---|---|---|
| **2009** | Guangdong | JB141090033/2009 | KC866919 | D2 | GenBank |
| **2009** | Guangdong | JB143090062/2008 | KC866903 | D2 | GenBank |
| **2009** | Guangdong | JB143090083/2009 | KC866917 | D2 | GenBank |
| **2009** | Guangdong | JB143090105/2008 | KC866902 | D2 | GenBank |
| **2009** | Guangdong | JB143090119/2009 | KC866921 | D2 | GenBank |
| **2009** | Guangdong | JB143090122/2009 | KC866916 | D2 | GenBank |
| **2009** | Guangdong | JB143090136/2009 | KC866906 | D2 | GenBank |
| **2009** | Guangdong | JB143090156/2009 | KC866914 | D2 | GenBank |
| **2009** | Guangdong | JB143090171/2009 | KC866913 | D2 | GenBank |
| **2009** | Guangdong | 2009-EV-106 | KM079505 | D3a | GenBank |
| **2009** | Guangdong | 2009-EV-22 | KM079504 | D3a | GenBank |
| **2009** | Guangdong | 2009-EV-233 | KM079506 | D3a | GenBank |
| **2009** | Guangdong | 2009-EV-235 | KM079507 | D3a | GenBank |
| **2009** | Guangdong | 2009-EV-260 | KM079508 | D3a | GenBank |
| **2009** | Guangdong | 2009-EV-261 | KM079509 | D3a | GenBank |
| **2009** | Guangdong | 2009-EV-334 | KM079510 | D3a | GenBank |
| **2009** | Guangdong | 2009-EV-363 | KM079511 | D3a | GenBank |
| **2009** | Guangdong | JB141090009/2009 | KC866918 | D3a | GenBank |
| **2009** | Guangdong | JB141090039/2009 | KC866905 | D3a | GenBank |
| **2009** | Guangdong | JB141090085/2009 | KC866912 | D3a | GenBank |
| **2009** | Guangdong | JB141090100/2009 | KC866907 | D3a | GenBank |
| **2009** | Guangdong | JB143090044/2009 | KC866908 | D3a | GenBank |
| **2009** | Guangdong | JB143090059/2009 | KC866909 | D3a | GenBank |
| **2009** | Guangdong | JB143090101/2009 | KC866904 | D3a | GenBank |
| **2009** | Guangdong | JB143090106/2009 | KC866920 | D3a | GenBank |
| **2009** | Guangdong | JB143090152/2009 | KC866910 | D3a | GenBank |
| **2009** | Japan | Kyoto3 | AB779615 | D1 | GenBank |
| **2009** | Japan | Kyoto4 | AB779617 | D1 | GenBank |
| **2009** | Taiwan | TW/20/2009 | JQ946050 | D3a | GenBank |
| **2010** | France | CF165026 | HE572917 | D1 | GenBank |
| **2010** | France | CF173039 | HE572923 | D1 | GenBank |
| **2010** | France | CF175080 | HE572930 | D1 | GenBank |
| **2010** | France | CF180026 | HE572932 | D1 | GenBank |
| **2010** | France | CF181032 | HE572935 | D1 | GenBank |
| **2010** | France | CF204023 | HE572939 | D1 | GenBank |
| **2010** | France | CF147014 | HE572909 | D3a | GenBank |
| **2010** | France | CF167084 | HE572920 | D3a | GenBank |
| **2010** | France | CF175076 | HE572928 | D3a | GenBank |
| **2010** | France | CF187035 | HE572936 | D3a | GenBank |
| **2010** | France | CF194028 | HE572938 | D3a | GenBank |
| **2010** | France | CF120091 | HE572903 | D3b | GenBank |
| **2010** | France | CF126012 | HE572904 | D3b | GenBank |
| **2010** | France | CF140007 | HE572906 | D3b | GenBank |
| **2010** | Guangdong | 109982/GZ/2010/A6 | KJ865427 | D2 | GenBank |

| | | | | | |
|---|---|---|---|---|---|
| **2010** | Guangdong | CVA6-SHZH2010-0601 | JX154921 | D2 | GenBank |
| **2010** | Guangdong | 1011374/GZ/2010/A6 | KJ865428 | D3a | GenBank |
| **2010** | Guangdong | 108721/GZ/2010/A6 | KJ865422 | D3a | GenBank |
| **2010** | Guangdong | 108867/GZ/2010/A6 | KJ865423 | D3a | GenBank |
| **2010** | Guangdong | 109097/GZ/2010/A6 | KJ865424 | D3a | GenBank |
| **2010** | Guangdong | 109223/GZ/2010/A6 | KJ865425 | D3a | GenBank |
| **2010** | Guangdong | 109833/GZ/2010/A6 | KJ865426 | D3a | GenBank |
| **2010** | Guangdong | 2010-EV-139 | KM079514 | D3a | GenBank |
| **2010** | Guangdong | 2010-EV-1559 | KM079524 | D3a | GenBank |
| **2010** | Guangdong | 2010-EV-311 | KM079515 | D3a | GenBank |
| **2010** | Guangdong | 2010-EV-944 | KM079516 | D3a | GenBank |
| **2010** | Guangdong | CVA6-SHZH2010-0903 | JX154929 | D3a | GenBank |
| **2010** | Guangdong | CVA6-SHZH2010-0906 | JX154932 | D3a | GenBank |
| **2010** | Guangdong | CVA6-SHZH2010-0907 | JX154933 | D3a | GenBank |
| **2010** | Japan | Hyogo5675 | LC126153 | D3b | GenBank |
| **2010** | Jiangsu | 10F84 Q3 2010 | KJ577273 | D2 | GenBank |
| **2010** | Jiangsu | 10MF64 Q4 2010 | KJ577274 | D3a | GenBank |
| **2010** | Jiangsu | 10MF66 Q4 2010 | KJ577275 | D3a | GenBank |
| **2010** | Jiangsu | 10MF67 Q4 2010 | KJ577276 | D3a | GenBank |
| **2010** | Jiangsu | 10MG26 Q4 2010 | KJ577277 | D3a | GenBank |
| **2010** | Jiangsu | 10MGJ34 Q2 2010 | KJ577278 | D3a | GenBank |
| **2010** | Jiangsu | 10MH26 Q4 2010 | KJ577279 | D3a | GenBank |
| **2010** | Shandong | 10032/SD/CHN/2010 | JQ364889 | D2 | GenBank |
| **2010** | Shanghai | 1232/SH/CHN/2010 | KJ541167 | D3a | GenBank |
| **2010** | Shanghai | SHAPHC1203/SH/CHN/10 | JX495122 | D3a | GenBank |
| **2010** | Shanghai | SHAPHC927/SH/CHN/10 | JX495124 | D3a | GenBank |
| **2010** | Taiwan | TW/409/2010 | JQ946055 | D3a | GenBank |
| **2011** | Japan | shizuoka 1 | AB649286 | D3b | GenBank |
| **2011** | Fujian | 2011FJQZ073 | KJ743209 | D2 | GenBank |
| **2011** | Fujian | 2011FJZZ156 | KJ743213 | D2 | GenBank |
| **2011** | Fujian | 2011FJLY035 | KJ743208 | D3a | GenBank |
| **2011** | Fujian | 2011FJQZ489 | KJ743210 | D3a | GenBank |
| **2011** | Fujian | 2011FJQZ495 | KJ743211 | D3a | GenBank |
| **2011** | Fujian | 2011FJZZ069 | KJ743212 | D3a | GenBank |
| **2011** | Gansu | 075/GS/CHN/2011 | KY211690 | D2 | this study |
| **2011** | Guangdong | 112614/GZ/2011/A6 | KJ865429 | D3a | GenBank |
| **2011** | Guangdong | 113549/GZ/2011/A6 | KJ865430 | D3a | GenBank |
| **2011** | Guangdong | 114089/GZ/2011/A6 | KJ865431 | D3a | GenBank |
| **2011** | Guangdong | 114361/GZ/2011/A6 | KJ865432 | D3a | GenBank |
| **2011** | Guangdong | 2011-EV-162 | KM079552 | D3a | GenBank |
| **2011** | Guangdong | 2011-EV-171 | KM079553 | D3a | GenBank |
| **2011** | Guangdong | 2011-EV-690 | KM079577 | D3a | GenBank |
| **2011** | Guangdong | 2011-EV-695 | KM079578 | D3a | GenBank |
| **2011** | Guangdong | CVA6-SHZH2011-0401 | JX473333 | D3a | GenBank |

| | | | | | |
|---|---|---|---|---|---|
| **2011** | Guangdong | CVA6-SHZH2011-0501 | JX473334 | D3a | GenBank |
| **2011** | Guangdong | CVA6-SHZH2011-1002 | JX473405 | D3a | GenBank |
| **2011** | Hebei | 54023/HeB/CHN/2011 | KY211707 | D2 | this study |
| **2011** | Henan | AYAYX11015/HN/CHN/2011 | KU708569 | D2 | GenBank |
| **2011** | Henan | AYLA11009/HN/CHN/2011 | KU708571 | D2 | GenBank |
| **2011** | Japan | Hyogo6427 | LC126155 | D3b | GenBank |
| **2011** | Japan | shizuoka 12 | AB649288 | D3b | GenBank |
| **2011** | Japan | shizuoka 19 | AB649291 | D3b | GenBank |
| **2011** | Jiangsu | 11MD3 Q1 2011 | KJ609186 | D2 | GenBank |
| **2011** | Jiangsu | 11MF21 Q2 2011 | KJ609187 | D2 | GenBank |
| **2011** | Jiangsu | 11MH15 Q2 2011 | KJ577297 | D2 | GenBank |
| **2011** | Jiangsu | 11MJ31 Q2 2011 | KJ577299 | D2 | GenBank |
| **2011** | Jiangsu | 11MA112 Q2 2011 | KJ577280 | D3a | GenBank |
| **2011** | Jiangsu | 11MA117 Q2 2011 | KJ577281 | D3a | GenBank |
| **2011** | Jiangsu | 11MF7 Q1 2011 | KJ609188 | D3a | GenBank |
| **2011** | Jiangsu | 11MG24 Q3 2011 | KJ577296 | D3a | GenBank |
| **2011** | Jiangsu | 11MH2 Q1 2011 | KJ577298 | D3a | GenBank |
| **2011** | Jiangsu | 11MJ32 Q3 2011 | KJ577300 | D3a | GenBank |
| **2011** | Shanghai | SHAPHC1883/SH/CHN/11 | JX495130 | D2 | GenBank |
| **2011** | Shanghai | SHAPHC2914/SH/CHN/11 | JX495118 | D2 | GenBank |
| **2011** | Shanghai | SHAPHC3012/SH/CHN/11 | JX495141 | D2 | GenBank |
| **2011** | Shanghai | 1827/SH/CHN/2011 | KJ541166 | D3a | GenBank |
| **2011** | Shanghai | 3913/SH/CHN/2011 | KJ541168 | D3a | GenBank |
| **2011** | Shanghai | SHAPHC1483/SH/CHN/11 | JX495121 | D3a | GenBank |
| **2011** | Shanghai | SHAPHC1575/SH/CHN/11 | JX495125 | D3a | GenBank |
| **2011** | Shanghai | SHAPHC1827/SH/CHN/11 | JX495123 | D3a | GenBank |
| **2011** | Shanghai | SHAPHC3455/SH/CHN/11 | JX495148 | D3a | GenBank |
| **2011** | Shanghai | SHAPHC3468/SH/CHN/11 | JX495149 | D3b | GenBank |
| **2011** | Shanxi | FH046/SX/CHN/2011 | KY211723 | D2 | this study |
| **2011** | Shanxi | FH058/SX/CHN/2011 | KY211724 | D2 | this study |
| **2011** | UK | V1/Ed/UK/2011 | KP144349 | D3 | GenBank |
| **2012** | Fujian | 2012FJFZ018 | KJ743214 | D3a | GenBank |
| **2012** | Fujian | 2012FJFZ177 | KJ743215 | D3a | GenBank |
| **2012** | Fujian | 2012FJFZ645 | KJ743216 | D3a | GenBank |
| **2012** | Fujian | 2012FJPT354 | KJ743231 | D3a | GenBank |
| **2012** | Fujian | 2012FJPT370 | KJ743232 | D3a | GenBank |
| **2012** | Fujian | 2012FJZZ512 | KJ743239 | D3a | GenBank |
| **2012** | Guangdong | 1212118/GZ/2012/A6 | KJ865448 | D3a | GenBank |
| **2012** | Guangdong | 1213442/GZ/2012/A6 | KJ865449 | D3a | GenBank |
| **2012** | Guangdong | 129994/GZ/2012/A6 | KJ865447 | D3a | GenBank |
| **2012** | Guangdong | 2012-EV-627 | KF836580 | D3a | GenBank |
| **2012** | Guangdong | 2012-EV-744 | KF836585 | D3a | GenBank |
| **2012** | Guangdong | 2012-EV-792 | KF836586 | D3a | GenBank |
| **2012** | Guangdong | 2012-EV-818 | KM079579 | D3a | GenBank |

| | | | | | |
|---|---|---|---|---|---|
| **2012** | Guangdong | 2012-EV-97 | KF836575 | D3a | GenBank |
| **2012** | Guangdong | JB141230260/2012 | KC866966 | D3a | GenBank |
| **2012** | Guangdong | 2012-EV-707 | KF836581 | D3b | GenBank |
| **2012** | Guangdong | 2012-EV-811 | KF836587 | D3b | GenBank |
| **2012** | Guangdong | JB141230253/2012 | KC866992 | D3b | GenBank |
| **2012** | Guangdong | JB141230261/2012 | KC866965 | D3b | GenBank |
| **2012** | Guangdong | JB141230262/2012 | KC866964 | D3b | GenBank |
| **2012** | Guangdong | JB141230296/2012 | KC867010 | D3b | GenBank |
| **2012** | Guangdong | JB141230301/2012 | KC866963 | D3b | GenBank |
| **2012** | Guangdong | JB141230314/2012 | KC866922 | D3b | GenBank |
| **2012** | Guangdong | JB141230315/2012 | KC866923 | D3b | GenBank |
| **2012** | Guangdong | JB141230316/2012 | KC866924 | D3b | GenBank |
| **2012** | Guangdong | JB141230319/2012 | KC866925 | D3b | GenBank |
| **2012** | Guangdong | JB141230329/2012 | KC866930 | D3b | GenBank |
| **2012** | Guangdong | JB141230336/2012 | KC866931 | D3b | GenBank |
| **2012** | Guangdong | JB141230340/2012 | KC866928 | D3b | GenBank |
| **2012** | Guangdong | JB141230344/2012 | KC866929 | D3b | GenBank |
| **2012** | Hebei | 54203/HeB/CHN/2012 | KY211711 | D2 | this study |
| **2012** | Henan | AYLA12156/HN/CHN/2012 | KU708572 | D2 | GenBank |
| **2012** | Henan | CS146/HN/CHN/12 | KJ156349 | D3a | GenBank |
| **2012** | Henan | CS577/HN/CHN/12 | KJ156351 | D3a | GenBank |
| **2012** | Jiangsu | 12MA194 Q4 2012 | KJ577302 | D3a | GenBank |
| **2012** | Jiangsu | 12MD45 Q4 2012 | KJ577304 | D3a | GenBank |
| **2012** | Jiangsu | 12MG39 Q4 2012 | KJ577309 | D3a | GenBank |
| **2012** | Jiangsu | 12MG41 Q4 2012 | KJ577310 | D3a | GenBank |
| **2012** | Jiangsu | 12MC46 Q4 2012 | KJ577303 | D3b | GenBank |
| **2012** | Shanghai | 4368/SH/CHN/2012 | KJ541169 | D3a | GenBank |
| **2012** | Shanghai | SHAPHC4493/SH/CHN/12 | KC414757 | D3a | GenBank |
| **2012** | Shanghai | SHAPHC4561/SH/CHN/12 | KC207832 | D3a | GenBank |
| **2012** | Shanghai | SHAPHC4689/SH/CHN/12 | KC414750 | D3a | GenBank |
| **2012** | Shanghai | SHAPHC4691/SH/CHN/12 | KC414751 | D3a | GenBank |
| **2012** | Shanghai | 4178/SH/CHN/2012 | KJ541439 | D3b | GenBank |
| **2012** | Shanghai | 4640/SH/CHN/2012 | KJ541438 | D3b | GenBank |
| **2012** | UK | V1/Ed/UK/2012 | KP144350 | D3a | GenBank |
| **2012** | UK | V13/Ed/UK/2012 | KP144341 | D3a | GenBank |
| **2012** | Xinjiang | 12-20/XJ/CHN/2012 | KY424426 | D3a | this study |
| **2012** | Xinjiang | 12-27/XJ/CHN/2012 | N/A | D3a | this study |
| **2012** | Xinjiang | 12-39/XJ/CHN/2012 | N/A | D3a | this study |
| **2012** | Xinjiang | 12-41/XJ/CHN/2012 | KY424425 | D3a | this study |
| **2013** | Chongqing | 13-2/CQ/CHN/2013 | N/A | D3a | this study |
| **2013** | Chongqing | 13-44/CQ/CHN/2013 | N/A | D3a | this study |
| **2013** | Chongqing | 13-7/CQ/CHN/2013 | N/A | D3a | this study |
| **2013** | Fujian | 2013FJQZ404 | KJ743243 | D3a | GenBank |
| **2013** | Gansu | 018/GS/CHN/2013 | KY211689 | D3a | this study |

| | | | | | |
|---|---|---|---|---|---|
| **2013** | Guangdong | 12743/GZ/CHN/2013 | KR815992 | D3a | GenBank |
| **2013** | Guangdong | 1320119/GZ/2013/A6 | KJ865461 | D3a | GenBank |
| **2013** | Guangdong | 1320770/GZ/2013/A6 | KJ865462 | D3a | GenBank |
| **2013** | Guangdong | 133702/GZ/2013/A6 | KJ865456 | D3a | GenBank |
| **2013** | Guangdong | 2013-CVA6-260 | KF734951 | D3a | GenBank |
| **2013** | Guangdong | 2013-CVA6-264 | KF734960 | D3a | GenBank |
| **2013** | Guangdong | 2013-CVA6-270 | KF734952 | D3a | GenBank |
| **2013** | Guangdong | 2013-CVA6-271 | KF734953 | D3a | GenBank |
| **2013** | Guangdong | 2013-CVA6-312 | KF734954 | D3a | GenBank |
| **2013** | Guangdong | 2013-CVA6-839 | KF734959 | D3a | GenBank |
| **2013** | Guangdong | 2013-EV-1152 | KF836570 | D3a | GenBank |
| **2013** | Guangdong | 2013-EV-1287 | KF836606 | D3a | GenBank |
| **2013** | Guangdong | 2013-EV-136 | KF836600 | D3a | GenBank |
| **2013** | Guangdong | 2013-EV-1523 | KM079584 | D3a | GenBank |
| **2013** | Guangdong | 2013-EV-1533 | KM079585 | D3a | GenBank |
| **2013** | Guangdong | 2013-EV-1534 | KM079586 | D3a | GenBank |
| **2013** | Guangdong | 2013-EV-1539 | KM079587 | D3a | GenBank |
| **2013** | Guangdong | 2013-EV-1545 | KM079588 | D3a | GenBank |
| **2013** | Guangdong | 2013-EV-350 | KF836548 | D3a | GenBank |
| **2013** | Guangdong | 2013-EV-376 | KF836549 | D3a | GenBank |
| **2013** | Guangdong | 2013-EV-38 | KF836594 | D3a | GenBank |
| **2013** | Guangdong | 2013-EV-57 | KF836595 | D3a | GenBank |
| **2013** | Guangdong | 2013-EV-870 | KF836555 | D3a | GenBank |
| **2013** | Guangdong | SHAPHC4978T/SH/CHN/13 | KF647886 | D3a | GenBank |
| **2013** | Guangdong | sz238A6/2013 | KJ018113 | D3a | GenBank |
| **2013** | Guangdong | sz269A6/2013 | KJ018114 | D3a | GenBank |
| **2013** | Guangdong | 2013-EV-4 | KF836593 | D3b | GenBank |
| **2013** | Guangdong | SHAPHC4792T/SH/CHN/13 | KF647876 | D3b | GenBank |
| **2013** | Guangdong | SHAPHC5084H/SH/CHN/13 | KF647892 | D3b | GenBank |
| **2013** | Guangdong | sz288A6/2013 | KJ018118 | D3b | GenBank |
| **2013** | Guizhou | 13-139/GZ/CHN/2013 | N/A | D3a | this study |
| **2013** | Guizhou | 13-140/GZ/CHN/2013 | N/A | D3a | this study |
| **2013** | Guizhou | 13-153/GZ/CHN/2013 | N/A | D3a | this study |
| **2013** | Guizhou | 13-157/GZ/CHN/2013 | N/A | D3a | this study |
| **2013** | Guizhou | 13-37/GZ/CHN/2013 | KY424405 | D3a | this study |
| **2013** | Guizhou | 13-42/GZ/CHN/2013 | KY424401 | D3a | this study |
| **2013** | Guizhou | 13-43/GZ/CHN/2013 | N/A | D3a | this study |
| **2013** | Guizhou | 13-44/GZ/CHN/2013 | N/A | D3a | this study |
| **2013** | Guizhou | 13-46/GZ/CHN/2013 | N/A | D3a | this study |
| **2013** | Guizhou | 13-62/GZ/CHN/2013 | N/A | D3a | this study |
| **2013** | Guizhou | 13-64/GZ/CHN/2013 | N/A | D3a | this study |
| **2013** | Guizhou | 13-7/GZ/CHN/2013 | KY424398 | D3a | this study |
| **2013** | Guizhou | 13-71/GZ/CHN/2013 | N/A | D3a | this study |
| **2013** | Guizhou | 13-73/GZ/CHN/2013 | N/A | D3a | this study |

| | | | | | |
|---|---|---|---|---|---|
| **2013** | Guizhou | 13-74/GZ/CHN/2013 | N/A | D3a | this study |
| **2013** | Guizhou | 13-77/GZ/CHN/2013 | N/A | D3a | this study |
| **2013** | Guizhou | 13-78/GZ/CHN/2013 | N/A | D3a | this study |
| **2013** | Hebei | 54011/HeB/CHN/2013 | N/A | D2 | this study |
| **2013** | Hebei | 54057/HeB/CHN/2013 | N/A | D2 | this study |
| **2013** | Hebei | 54090/HeB/CHN/2013 | N/A | D2 | this study |
| **2013** | Hebei | 54103/HeB/CHN/2013 | N/A | D2 | this study |
| **2013** | Hebei | 54107/HeB/CHN/2013 | N/A | D2 | this study |
| **2013** | Hebei | 54001/HeB/CHN/2013 | KY211706 | D3a | this study |
| **2013** | Hebei | 54021/HeB/CHN/2013 | N/A | D3a | this study |
| **2013** | Hebei | 54083/HeB/CHN/2013 | KY211708 | D3a | this study |
| **2013** | Hebei | 54149/HeB/CHN/2013 | KY211710 | D3a | this study |
| **2013** | Hebei | 54058/HeB/CHN/2013 | N/A | D3b | this study |
| **2013** | Hebei | 54106/HeB/CHN/2013 | KY424359 | D3b | this study |
| **2013** | Henan | 13-100/HeN/CHN/2013 | N/A | D2 | this study |
| **2013** | Henan | 13-110/HeN/CHN/2013 | KY424419 | D2 | this study |
| **2013** | Henan | 13-32/HeN/CHN/2013 | N/A | D2 | this study |
| **2013** | Henan | 13-63/HeN/CHN/2013 | N/A | D2 | this study |
| **2013** | Henan | 13-71/HeN/CHN/2013 | N/A | D2 | this study |
| **2013** | Henan | 13-9/HeN/CHN/2013 | KY424393 | D2 | this study |
| **2013** | Henan | AYLA13003/HN/CHN/2013 | KU708574 | D2 | GenBank |
| **2013** | Henan | AYLA13048/HN/CHN/2013 | KT124601 | D2 | GenBank |
| **2013** | Henan | AYLA13050/HN/CHN/2013 | KT124602 | D2 | GenBank |
| **2013** | Henan | 13-103/HeN/CHN/2013 | KY424422 | D3a | this study |
| **2013** | Henan | 13-119/HeN/CHN/2013 | KY424416 | D3a | this study |
| **2013** | Henan | 13-120/HeN/CHN/2013 | N/A | D3a | this study |
| **2013** | Henan | 13-121/HeN/CHN/2013 | N/A | D3a | this study |
| **2013** | Henan | 13-122/HeN/CHN/2013 | N/A | D3a | this study |
| **2013** | Henan | 13-128/HeN/CHN/2013 | N/A | D3a | this study |
| **2013** | Henan | 13-130/HeN/CHN/2013 | N/A | D3a | this study |
| **2013** | Henan | 13-17/HeN/CHN/2013 | N/A | D3a | this study |
| **2013** | Henan | 13-19/HeN/CHN/2013 | N/A | D3a | this study |
| **2013** | Henan | 13-26/HeN/CHN/2013 | KY424410 | D3a | this study |
| **2013** | Henan | 13-3/HeN/CHN/2013 | N/A | D3a | this study |
| **2013** | Henan | 13-30/HeN/CHN/2013 | N/A | D3a | this study |
| **2013** | Henan | 13-31/HeN/CHN/2013 | N/A | D3a | this study |
| **2013** | Henan | 13-34/HeN/CHN/2013 | N/A | D3a | this study |
| **2013** | Henan | 13-36/HeN/CHN/2013 | N/A | D3a | this study |
| **2013** | Henan | 13-38/HeN/CHN/2013 | KY424403 | D3a | this study |
| **2013** | Henan | 13-40/HeN/CHN/2013 | KY424402 | D3a | this study |
| **2013** | Henan | 13-41/HeN/CHN/2013 | N/A | D3a | this study |
| **2013** | Henan | 13-46/HeN/CHN/2013 | N/A | D3a | this study |
| **2013** | Henan | 13-51/HeN/CHN/2013 | KY424399 | D3a | this study |
| **2013** | Henan | 13-52/HeN/CHN/2013 | N/A | D3a | this study |

| | | | | | |
|---|---|---|---|---|---|
| **2013** | Henan | 13-53/HeN/CHN/2013 | N/A | D3a | this study |
| **2013** | Henan | 13-54/HeN/CHN/2013 | N/A | D3a | this study |
| **2013** | Henan | 13-56/HeN/CHN/2013 | N/A | D3a | this study |
| **2013** | Henan | 13-57/HeN/CHN/2013 | N/A | D3a | this study |
| **2013** | Henan | 13-58/HeN/CHN/2013 | N/A | D3a | this study |
| **2013** | Henan | 13-59/HeN/CHN/2013 | N/A | D3a | this study |
| **2013** | Henan | 13-60/HeN/CHN/2013 | N/A | D3a | this study |
| **2013** | Henan | 13-61/HeN/CHN/2013 | N/A | D3a | this study |
| **2013** | Henan | 13-62/HeN/CHN/2013 | N/A | D3a | this study |
| **2013** | Henan | 13-67/HeN/CHN/2013 | N/A | D3a | this study |
| **2013** | Henan | 13-68/HeN/CHN/2013 | N/A | D3a | this study |
| **2013** | Henan | 13-81/HeN/CHN/2013 | KY424395 | D3a | this study |
| **2013** | Henan | 13-82/HeN/CHN/2013 | N/A | D3a | this study |
| **2013** | Henan | 13-88/HeN/CHN/2013 | N/A | D3a | this study |
| **2013** | Henan | 13-93/HeN/CHN/2013 | N/A | D3a | this study |
| **2013** | Henan | AYLZ13014/HN/CHN/2013 | KU708616 | D3a | GenBank |
| **2013** | India | CVA6/MCVR/7007 | KU366286 | D3a | GenBank |
| **2013** | India | CVA6/MCVR/7385 | KU366287 | D3a | GenBank |
| **2013** | Japan | Hyogo9205 | LC126161 | D3b | GenBank |
| **2013** | Japan | Hyogo9288 | LC126162 | D3b | GenBank |
| **2013** | Jiangsu | 13MA218 Q1 2013 | KJ577316 | D3a | GenBank |
| **2013** | Jiangsu | 13MA219 Q2 2013 | KJ577317 | D3a | GenBank |
| **2013** | Jiangsu | 13MA220 Q2 2013 | KJ577318 | D3a | GenBank |
| **2013** | Jiangsu | 13MA245 Q3 2013 | KJ577325 | D3a | GenBank |
| **2013** | Jiangsu | 13MA252 Q4 2013 | KJ577330 | D3a | GenBank |
| **2013** | Jiangsu | 13MA256 Q4 2013 | KJ577331 | D3a | GenBank |
| **2013** | Jiangsu | 13MC24 Q2 2013 | KJ609189 | D3a | GenBank |
| **2013** | Jiangsu | 13MC27 Q2 2013 | KJ577345 | D3a | GenBank |
| **2013** | Jiangsu | 13MF22 Q2 2013 | KJ609193 | D3a | GenBank |
| **2013** | Jiangsu | 13MFJ4 Q2 2013 | KJ577377 | D3a | GenBank |
| **2013** | Jiangxi | 13-100/JX/CHN/2013 | KY424423 | D3a | this study |
| **2013** | Jiangxi | 13-106/JX/CHN/2013 | N/A | D3a | this study |
| **2013** | Jiangxi | 13-110/JX/CHN/2013 | KY424418 | D3a | this study |
| **2013** | Jiangxi | 13-113/JX/CHN/2013 | KY424417 | D3a | this study |
| **2013** | Jiangxi | 13-118/JX/CHN/2013 | N/A | D3a | this study |
| **2013** | Jiangxi | 13-124/JX/CHN/2013 | N/A | D3a | this study |
| **2013** | Jiangxi | 13-13/JX/CHN/2013 | N/A | D3a | this study |
| **2013** | Jiangxi | 13-18/JX/CHN/2013 | N/A | D3a | this study |
| **2013** | Jiangxi | 13-22/JX/CHN/2013 | N/A | D3a | this study |
| **2013** | Jiangxi | 13-25/JX/CHN/2013 | N/A | D3a | this study |
| **2013** | Jiangxi | 13-35/JX/CHN/2013 | N/A | D3a | this study |
| **2013** | Jiangxi | 13-43/JX/CHN/2013 | N/A | D3a | this study |
| **2013** | Jiangxi | 13-44/JX/CHN/2013 | N/A | D3a | this study |
| **2013** | Jiangxi | 13-53/JX/CHN/2013 | N/A | D3a | this study |

| | | | | | |
|---|---|---|---|---|---|
| **2013** | Jiangxi | 13-59/JX/CHN/2013 | N/A | D3a | this study |
| **2013** | Jiangxi | 13-60/JX/CHN/2013 | N/A | D3a | this study |
| **2013** | Jiangxi | 13-63/JX/CHN/2013 | N/A | D3a | this study |
| **2013** | Jiangxi | 13-71/JX/CHN/2013 | N/A | D3a | this study |
| **2013** | Jiangxi | 13-75/JX/CHN/2013 | N/A | D3a | this study |
| **2013** | Jiangxi | 13-76/JX/CHN/2013 | N/A | D3a | this study |
| **2013** | Jiangxi | 13-78/JX/CHN/2013 | N/A | D3a | this study |
| **2013** | Jiangxi | 13-82/JX/CHN/2013 | N/A | D3a | this study |
| **2013** | Jiangxi | 13-83/JX/CHN/2013 | N/A | D3a | this study |
| **2013** | Jiangxi | 13-90/JX/CHN/2013 | N/A | D3a | this study |
| **2013** | Jiangxi | 13-93/JX/CHN/2013 | N/A | D3a | this study |
| **2013** | Jiangxi | 13-95/JX/CHN/2013 | KY424391 | D3a | this study |
| **2013** | Jiangxi | 13-98/JX/CHN/2013 | KY424389 | D3a | this study |
| **2013** | Jilin | 13-40/JL/CHN/2013 | N/A | D2 | this study |
| **2013** | Jilin | 13-97/JL/CHN/2013 | KY424390 | D2 | this study |
| **2013** | Jilin | 13-98/JL/CHN/2013 | N/A | D2 | this study |
| **2013** | Jilin | 13-100/JL/CHN/2013 | N/A | D3a | this study |
| **2013** | Jilin | 13-101/JL/CHN/2013 | N/A | D3a | this study |
| **2013** | Jilin | 13-102/JL/CHN/2013 | N/A | D3a | this study |
| **2013** | Jilin | 13-106/JL/CHN/2013 | N/A | D3a | this study |
| **2013** | Jilin | 13-107/JL/CHN/2013 | KY424420 | D3a | this study |
| **2013** | Jilin | 13-108/JL/CHN/2013 | N/A | D3a | this study |
| **2013** | Jilin | 13-27/JL/CHN/2013 | N/A | D3a | this study |
| **2013** | Jilin | 13-43/JL/CHN/2013 | N/A | D3a | this study |
| **2013** | Jilin | 13-57/JL/CHN/2013 | N/A | D3a | this study |
| **2013** | Jilin | 13-61/JL/CHN/2013 | N/A | D3a | this study |
| **2013** | Jilin | 13-62/JL/CHN/2013 | N/A | D3a | this study |
| **2013** | Jilin | 13-63/JL/CHN/2013 | N/A | D3a | this study |
| **2013** | Jilin | 13-64/JL/CHN/2013 | N/A | D3a | this study |
| **2013** | Jilin | 13-67/JL/CHN/2013 | N/A | D3a | this study |
| **2013** | Jilin | 13-96/JL/CHN/2013 | N/A | D3a | this study |
| **2013** | Jilin | 13-99/JL/CHN/2013 | N/A | D3a | this study |
| **2013** | Liaoning | 13-104/LN/CHN/2013 | KY424421 | D3a | this study |
| **2013** | Shaanxi | 13-17/SaX/CHN/2013 | N/A | D2 | this study |
| **2013** | Shaanxi | 13-3/SaX/CHN/2013 | KY424408 | D2 | this study |
| **2013** | Shaanxi | 13-106/SaX/CHN/2013 | N/A | D3a | this study |
| **2013** | Shaanxi | 13-110/SaX/CHN/2013 | N/A | D3a | this study |
| **2013** | Shaanxi | 13-12/SaX/CHN/2013 | N/A | D3a | this study |
| **2013** | Shaanxi | 13-124/SaX/CHN/2013 | N/A | D3a | this study |
| **2013** | Shaanxi | 13-126/SaX/CHN/2013 | N/A | D3a | this study |
| **2013** | Shaanxi | 13-13/SaX/CHN/2013 | N/A | D3a | this study |
| **2013** | Shaanxi | 13-130/SaX/CHN/2013 | N/A | D3a | this study |
| **2013** | Shaanxi | 13-132/SaX/CHN/2013 | N/A | D3a | this study |
| **2013** | Shaanxi | 13-140/SaX/CHN/2013 | KY424415 | D3a | this study |

| | | | | | |
|---|---|---|---|---|---|
| **2013** | Shaanxi | 13-144/SaX/CHN/2013 | N/A | D3a | this study |
| **2013** | Shaanxi | 13-16/SaX/CHN/2013 | N/A | D3a | this study |
| **2013** | Shaanxi | 13-19/SaX/CHN/2013 | N/A | D3a | this study |
| **2013** | Shaanxi | 13-20/SaX/CHN/2013 | N/A | D3a | this study |
| **2013** | Shaanxi | 13-23/SaX/CHN/2013 | N/A | D3a | this study |
| **2013** | Shaanxi | 13-27/SaX/CHN/2013 | N/A | D3a | this study |
| **2013** | Shaanxi | 13-30/SaX/CHN/2013 | N/A | D3a | this study |
| **2013** | Shaanxi | 13-31/SaX/CHN/2013 | N/A | D3a | this study |
| **2013** | Shaanxi | 13-37/SaX/CHN/2013 | N/A | D3a | this study |
| **2013** | Shaanxi | 13-4/SaX/CHN/2013 | N/A | D3a | this study |
| **2013** | Shaanxi | 13-45/SaX/CHN/2013 | KY424400 | D3a | this study |
| **2013** | Shaanxi | 13-47/SaX/CHN/2013 | N/A | D3a | this study |
| **2013** | Shaanxi | 13-49/SaX/CHN/2013 | N/A | D3a | this study |
| **2013** | Shaanxi | 13-5/SaX/CHN/2013 | N/A | D3a | this study |
| **2013** | Shaanxi | 13-54/SaX/CHN/2013 | N/A | D3a | this study |
| **2013** | Shaanxi | 13-59/SaX/CHN/2013 | N/A | D3a | this study |
| **2013** | Shaanxi | 13-6/SaX/CHN/2013 | N/A | D3a | this study |
| **2013** | Shaanxi | 13-60/SaX/CHN/2013 | N/A | D3a | this study |
| **2013** | Shaanxi | 13-67/SaX/CHN/2013 | N/A | D3a | this study |
| **2013** | Shaanxi | 13-70/SaX/CHN/2013 | N/A | D3a | this study |
| **2013** | Shaanxi | 13-71/SaX/CHN/2013 | N/A | D3a | this study |
| **2013** | Shaanxi | 13-72/SaX/CHN/2013 | N/A | D3a | this study |
| **2013** | Shaanxi | 13-73/SaX/CHN/2013 | N/A | D3a | this study |
| **2013** | Shaanxi | 13-74/SaX/CHN/2013 | KY424396 | D3a | this study |
| **2013** | Shaanxi | 13-76/SaX/CHN/2013 | N/A | D3a | this study |
| **2013** | Shaanxi | 13-78/SaX/CHN/2013 | N/A | D3a | this study |
| **2013** | Shaanxi | 13-79/SaX/CHN/2013 | N/A | D3a | this study |
| **2013** | Shaanxi | 13-8/SaX/CHN/2013 | N/A | D3a | this study |
| **2013** | Shaanxi | 13-80/SaX/CHN/2013 | N/A | D3a | this study |
| **2013** | Shaanxi | 13-81/SaX/CHN/2013 | N/A | D3a | this study |
| **2013** | Shaanxi | 13-82/SaX/CHN/2013 | N/A | D3a | this study |
| **2013** | Shaanxi | 13-83/SaX/CHN/2013 | N/A | D3a | this study |
| **2013** | Shaanxi | 13-89/SaX/CHN/2013 | N/A | D3a | this study |
| **2013** | Shaanxi | 13-9/SaX/CHN/2013 | KY424392 | D3a | this study |
| **2013** | Shaanxi | 13-90/SaX/CHN/2013 | N/A | D3a | this study |
| **2013** | Shaanxi | 13-93/SaX/CHN/2013 | N/A | D3a | this study |
| **2013** | Shaanxi | 13-94/SaX/CHN/2013 | N/A | D3a | this study |
| **2013** | Shaanxi | 13-95/SaX/CHN/2013 | N/A | D3a | this study |
| **2013** | Shaanxi | 13-96/SaX/CHN/2013 | N/A | D3a | this study |
| **2013** | Shaanxi | 13-99/SaX/CHN/2013 | KY424388 | D3a | this study |
| **2013** | Shaanxi | 13-75/SaX/CHN/2013 | N/A | D3b | this study |
| **2013** | Shaanxi | 13-87/SaX/CHN/2013 | KY424394 | D3b | this study |
| **2013** | Shandong | 101/SZK107/SD/CHN/2013 | KY211691 | D3a | this study |
| **2013** | Shandong | 127/HF53/SD/CHN/2013 | KY211693 | D3a | this study |

| | | | | | |
|---|---|---|---|---|---|
| **2013** | Shandong | 219LW03/SD/CHN/2013 | N/A | D3a | this study |
| **2013** | Shandong | 230/ZZ177/SD/CHN/2013 | KY211701 | D3a | this study |
| **2013** | Shandong | 243DY003/SD/CHN/2013 | N/A | D3a | this study |
| **2013** | Shandong | 245/DY005/SD/CHN/2013 | KY211702 | D3a | this study |
| **2013** | Shanghai | 4645/SH/CHN/2012 | KJ541157 | D3a | GenBank |
| **2013** | Shanghai | 4937/SH/CHN/2013 | KJ541441 | D3a | GenBank |
| **2013** | Shanghai | 4940/SH/CHN/2013 | KJ541440 | D3a | GenBank |
| **2013** | Shanghai | 5035/SH/CHN/2013 | KJ541436 | D3a | GenBank |
| **2013** | Shanghai | 5039/SH/CHN/2013 | KJ541160 | D3a | GenBank |
| **2013** | Shanghai | 5056/SH/CHN/2013 | KJ541161 | D3a | GenBank |
| **2013** | Shanghai | 5069/SH/CHN/2013 | KJ541158 | D3a | GenBank |
| **2013** | Shanghai | 5103/SH/CHN/2013 | KJ541437 | D3a | GenBank |
| **2013** | Shanghai | PF10/SH/CHN/2013 | KJ541368 | D3a | GenBank |
| **2013** | Shanghai | PF29/SH/CHN/2013 | KJ541371 | D3a | GenBank |
| **2013** | Shanghai | PF3/SH/CHN/2013 | KJ541162 | D3a | GenBank |
| **2013** | Shanghai | PF4/SH/CHN/2013 | KJ541370 | D3a | GenBank |
| **2013** | Shanghai | PF9/SH/CHN/2013 | KJ541369 | D3a | GenBank |
| **2013** | Shanghai | 5047/SH/CHN/2013 | KJ541156 | D3b | GenBank |
| **2013** | Shanghai | 5084/SH/CHN/2013 | KJ541154 | D3b | GenBank |
| **2013** | Shanghai | PF001/SH/CHN/2013 | KJ541165 | D3b | GenBank |
| **2013** | Shanghai | PF19/SH/CHN/2013 | KJ541155 | D3b | GenBank |
| **2013** | Shanghai | PF19/SH/CHN/2013 | KJ541385 | D3b | GenBank |
| **2013** | Shanxi | FH233/SX/CHN/2013 | KY211727 | D2 | this study |
| **2013** | Shanxi | FH216/SX/CHN/2013 | KY211726 | D3a | this study |
| **2013** | Shanxi | FH239/SX/CHN/2013 | KY211727 | D3a | this study |
| **2013** | Shanxi | FH117/SX/CHN/2013 | KY211725 | D3b | this study |
| **2013** | Sichuan | 13-08/SC/CHN/2013 | KY424424 | D3a | this study |
| **2013** | Sichuan | 13-21/SC/CHN/2013 | KY424414 | D3a | this study |
| **2013** | Sichuan | 13-36/SC/CHN/2013 | N/A | D3a | this study |
| **2013** | Sichuan | 13-37/SC/CHN/2013 | N/A | D3a | this study |
| **2013** | Sichuan | 13-41/SC/CHN/2013 | N/A | D3a | this study |
| **2013** | Tianjin | TJ13-91-C-CVA6 | KJ848296 | D2 | GenBank |
| **2013** | Tianjin | TJ13-13-C-CVA6 | KJ848309 | D3a | GenBank |
| **2013** | Tianjin | TJ13-45-C-CVA6 | KJ848305 | D3a | GenBank |
| **2013** | Tianjin | TJ13-54-C-CVA6 | KJ848308 | D3a | GenBank |
| **2013** | Tianjin | TJ13-68-C-CVA6 | KJ848323 | D3a | GenBank |
| **2013** | Tianjin | TJ13-69-C-CVA6 | KJ848307 | D3a | GenBank |
| **2013** | Tianjin | TJ13-79-C-CVA6 | KJ848324 | D3a | GenBank |
| **2013** | Tianjin | TJ13-85-C-CVA6 | KJ848306 | D3a | GenBank |
| **2013** | UK | V13/Ed/UK/2013 | KP144339 | D3a | GenBank |
| **2013** | Xinjiang | 13-31/XJ/CHN/2013 | N/A | D2 | this study |
| **2013** | Xinjiang | 13-34/XJ/CHN/2013 | KY424406 | D2 | this study |
| **2013** | Xinjiang | 13-21/XJ/CHN/2013 | KY424413 | D3a | this study |
| **2013** | Xinjiang | 13-22/XJ/CHN/2013 | N/A | D3a | this study |

| | | | | | |
|---|---|---|---|---|---|
| **2013** | Xinjiang | 13-26/XJ/CHN/2013 | N/A | D3a | this study |
| **2013** | Xinjiang | 13-32/XJ/CHN/2013 | KY424407 | D3a | this study |
| **2013** | Xinjiang | 13-37/XJ/CHN/2013 | KY424404 | D3a | this study |
| **2013** | Xinjiang | 13-40/XJ/CHN/2013 | N/A | D3a | this study |
| **2013** | Xinjiang | 13-41/XJ/CHN/2013 | N/A | D3a | this study |
| **2013** | Xinjiang | 13-45/XJ/CHN/2013 | N/A | D3a | this study |
| **2013** | Xinjiang | 13-47/XJ/CHN/2013 | N/A | D3a | this study |
| **2013** | Yunnan | A023/YN/CHN/2013 | KY424358 | D2 | this study |
| **2013** | Yunnan | A004/YN/CHN/2013 | N/A | D3a | this study |
| **2013** | Yunnan | A017/YN/CHN/2013 | N/A | D3a | this study |
| **2013** | Yunnan | A036/YN/CHN/2013 | N/A | D3a | this study |
| **2013** | Yunnan | A065/YN/CHN/2013 | N/A | D3a | this study |
| **2013** | Yunnan | A144/YN/CHN/2013 | KY211715 | D3a | this study |
| **2013** | Yunnan | K12/YN/CHN/2013 | N/A | D3a | this study |
| **2013** | Yunnan | K8/YN/CHN/2013 | N/A | D3a | this study |
| **2013** | Yunnan | K9/YN/CHN/2013 | N/A | D3a | this study |
| **2013** | Yunnan | N26/YN/CHN/2013 | N/A | D3a | this study |
| **2013** | Yunnan | N27/YN/CHN/2013 | KY211736 | D3a | this study |
| **2013** | Yunnan | N34/YN/CHN/2013 | KY211737 | D3a | this study |
| **2013** | Zhejiang | 13-17/ZJ/CHN/2013 | N/A | D3a | this study |
| **2013** | Zhejiang | 13-20/ZJ/CHN/2013 | N/A | D3a | this study |
| **2013** | Zhejiang | 13-21/ZJ/CHN/2013 | KY424412 | D3a | this study |
| **2013** | Zhejiang | 13-24/ZJ/CHN/2013 | KY424411 | D3a | this study |
| **2013** | Zhejiang | 13-25/ZJ/CHN/2013 | N/A | D3a | this study |
| **2013** | Zhejiang | 13-26/ZJ/CHN/2013 | N/A | D3a | this study |
| **2013** | Zhejiang | 13-27/ZJ/CHN/2013 | N/A | D3a | this study |
| **2013** | Zhejiang | 13-28/ZJ/CHN/2013 | KY424409 | D3a | this study |
| **2013** | Zhejiang | 13-40/ZJ/CHN/2013 | N/A | D3a | this study |
| **2013** | Zhejiang | 13-42/ZJ/CHN/2013 | N/A | D3a | this study |
| **2013** | Zhejiang | 13-48/ZJ/CHN/2013 | N/A | D3a | this study |
| **2013** | Zhejiang | 13-49/ZJ/CHN/2013 | N/A | D3a | this study |
| **2013** | Zhejiang | 13-55/ZJ/CHN/2013 | N/A | D3a | this study |
| **2013** | Zhejiang | 13-57/ZJ/CHN/2013 | N/A | D3a | this study |
| **2013** | Zhejiang | 13-7/ZJ/CHN/2013 | KY424397 | D3a | this study |
| **2013** | Zhejiang | CV-A6/P289/2013/China | KP289365 | D3a | GenBank |
| **2013** | Zhejiang | CV-A6/P358/2013/China | KP289377 | D3a | GenBank |
| **2013** | Zhejiang | CV-A6/P360/2013/China | KP289378 | D3a | GenBank |
| **2013** | Zhejiang | CV-A6/P361/2013/China | KP289379 | D3a | GenBank |
| **2013** | Zhejiang | CV-A6/P423/2013/China | KP289366 | D3a | GenBank |
| **2013** | Zhejiang | CV-A6/P426/2013/China | KP289383 | D3a | GenBank |
| **2013** | Zhejiang | CV-A6/P874/2013/China | KP289393 | D3a | GenBank |
| **2014** | Gansu | 1010/GS/CHN/2014 | KY211692 | D3a | this study |
| **2014** | Gansu | 867/GS/CHN/2014 | KY211713 | D3a | this study |
| **2014** | Henan | AYLA14083/HN/CHN/2014 | KU708581 | D3a | GenBank |

| | | | | | |
|---|---|---|---|---|---|
| **2014** | Henan | AYLA14083/HN/CHN/2014 | KU708581 | D3a | GenBank |
| **2014** | Henan | AYLA14144/HN/CHN/2014 | KU708583 | D3a | GenBank |
| **2014** | Henan | AYLA14232/HN/CHN/2014 | KU708584 | D3a | GenBank |
| **2014** | Henan | AYLA14258/HN/CHN/2014 | KU708585 | D3a | GenBank |
| **2014** | Henan | AYLA14292/HN/CHN/2014 | KU708586 | D3a | GenBank |
| **2014** | Henan | AYLA14369/HN/CHN/2014 | KU708590 | D3a | GenBank |
| **2014** | Henan | AYLA14385/HN/CHN/2014 | KU708591 | D3a | GenBank |
| **2014** | Henan | AYLA14385/HN/CHN/2014 | KU708591 | D3a | GenBank |
| **2014** | Henan | AYLA14399/HN/CHN/2014 | KU708592 | D3a | GenBank |
| **2014** | Henan | AYNH14030/HN/CHN/2014 | KU708618 | D3a | GenBank |
| **2014** | Henan | AYNH14030/HN/CHN/2014 | KU708618 | D3a | GenBank |
| **2014** | Hunan | 14-105/HuN/CHN/2014 | N/A | D3a | this study |
| **2014** | Hunan | 14-106/HuN/CHN/2014 | KY424386 | D3a | this study |
| **2014** | Hunan | 14-108/HuN/CHN/2014 | KY424384 | D3a | this study |
| **2014** | Hunan | 14-110/HuN/CHN/2014 | N/A | D3a | this study |
| **2014** | Hunan | 14-112/HuN/CHN/2014 | N/A | D3a | this study |
| **2014** | Hunan | 14-77/HuN/CHN/2014 | N/A | D3a | this study |
| **2014** | Hunan | 14-91/HuN/CHN/2014 | N/A | D3a | this study |
| **2014** | Hunan | 14-98/HuN/CHN/2014 | KY424373 | D3a | this study |
| **2014** | Jiangxi | 14-106/JX/CHN/2014 | KY424385 | D3a | this study |
| **2014** | Jiangxi | 14-111/JX/CHN/2014 | KY424383 | D3a | this study |
| **2014** | Jiangxi | 14-118/JX/CHN/2014 | N/A | D3a | this study |
| **2014** | Jiangxi | 14-123/JX/CHN/2014 | N/A | D3a | this study |
| **2014** | Jiangxi | 14-124/JX/CHN/2014 | N/A | D3a | this study |
| **2014** | Jiangxi | 14-126/JX/CHN/2014 | N/A | D3a | this study |
| **2014** | Jiangxi | 14-16/JX/CHN/2014 | N/A | D3a | this study |
| **2014** | Jiangxi | 14-4/JX/CHN/2014 | N/A | D3a | this study |
| **2014** | Jiangxi | 14-66/JX/CHN/2014 | KY424380 | D3a | this study |
| **2014** | Jiangxi | 14-75/JX/CHN/2014 | KY424379 | D3a | this study |
| **2014** | Jiangxi | 14-94/JX/CHN/2014 | KY424375 | D3a | this study |
| **2014** | Liaoning | 14-233/LN/CHN/2014 | KY424382 | D3a | this study |
| **2014** | Shaanxi | 14-1/SaX/CHN/2014 | KY424387 | D3a | this study |
| **2014** | Shaanxi | 14-26/SaX/CHN/2014 | KY424381 | D3a | this study |
| **2014** | Shandong | 70LY100/SD/CHN/2014 | N/A | D3a | this study |
| **2014** | Shanghai | SHAPHC5219/SH/CHN/14 | KU212261 | D3a | GenBank |
| **2014** | Shanghai | SHAPHC5221/SH/CHN/14 | KU212263 | D3a | GenBank |
| **2014** | Shanghai | SHAPHC5229/SH/CHN/14 | KU212264 | D3a | GenBank |
| **2014** | Shanghai | SHAPHC5265/SH/CHN/14 | KU212262 | D3a | GenBank |
| **2014** | Shanghai | SHAPHC5444/SH/CHN/14 | KU212267 | D3a | GenBank |
| **2014** | Shanghai | SHAPHC5494/SH/CHN/14 | KU212268 | D3a | GenBank |
| **2014** | Shanghai | SHAPHC5518/SH/CHN/14 | KU212272 | D3a | GenBank |
| **2014** | Shanghai | SHAPHC5524/SH/CHN/14 | KU212273 | D3a | GenBank |
| **2014** | Shanghai | SHAPHC5530/SH/CHN/14 | KU212265 | D3a | GenBank |
| **2014** | Shanghai | SHAPHC5547/SH/CHN/14 | KU212269 | D3a | GenBank |

| | | | | | |
|---|---|---|---|---|---|
| **2014** | Shanghai | SHAPHC5550/SH/CHN/14 | KU212266 | D3a | GenBank |
| **2014** | Shanghai | SHAPHC5570/SH/CHN/14 | KU212271 | D3a | GenBank |
| **2014** | Shanghai | SHAPHC5588/SH/CHN/14 | KU212270 | D3a | GenBank |
| **2014** | Shanghai | SHAPHC5606/SH/CHN/14 | KU212274 | D3a | GenBank |
| **2014** | Shanghai | SHAPHC5619/SH/CHN/14 | KU212275 | D3a | GenBank |
| **2014** | Sichuan | 14-88/SC/CHN/2014 | KY424376 | D3a | this study |
| **2014** | Sichuan | 14-89/SC/CHN/2014 | N/A | D3a | this study |
| **2014** | UK | V4/Ed/UK/2014 | KP144348 | D3a | GenBank |
| **2014** | UK | V1/Gla/UK/2014 | KP144343 | D3b | GenBank |
| **2014** | Xinjiang | 14-85/XJ/CHN/2014 | N/A | D2 | this study |
| **2014** | Xinjiang | 14-94/XJ/CHN/2014 | KY424374 | D2 | this study |
| **2014** | Xinjiang | 14-62/XJ/CHN/2014 | N/A | D3a | this study |
| **2014** | Xinjiang | 14-63/XJ/CHN/2014 | N/A | D3a | this study |
| **2014** | Xinjiang | 14-65/XJ/CHN/2014 | N/A | D3a | this study |
| **2014** | Xinjiang | 14-67/XJ/CHN/2014 | N/A | D3a | this study |
| **2014** | Xinjiang | 14-68/XJ/CHN/2014 | N/A | D3a | this study |
| **2014** | Xinjiang | 14-70/XJ/CHN/2014 | N/A | D3a | this study |
| **2014** | Xinjiang | 14-72/XJ/CHN/2014 | N/A | D3a | this study |
| **2014** | Xinjiang | 14-73/XJ/CHN/2014 | N/A | D3a | this study |
| **2014** | Xinjiang | 14-74/XJ/CHN/2014 | N/A | D3a | this study |
| **2014** | Xinjiang | 14-77/XJ/CHN/2014 | KY424378 | D3a | this study |
| **2014** | Xinjiang | 14-81/XJ/CHN/2014 | KY424377 | D3a | this study |
| **2014** | Xinjiang | 14-82/XJ/CHN/2014 | N/A | D3a | this study |
| **2014** | Xinjiang | 14-86/XJ/CHN/2014 | N/A | D3a | this study |
| **2014** | Xinjiang | 14-87/XJ/CHN/2014 | N/A | D3a | this study |
| **2014** | Xinjiang | 14-89/XJ/CHN/2014 | N/A | D3a | this study |
| **2014** | Xinjiang | 14-92/XJ/CHN/2014 | N/A | D3a | this study |
| **2014** | Xinjiang | 14-93/XJ/CHN/2014 | N/A | D3a | this study |
| **2014** | Xinjiang | 14-95/XJ/CHN/2014 | N/A | D3a | this study |
| **2014** | Yunnan | A145/YN/CHN/2014 | N/A | D3a | this study |
| **2014** | Yunnan | A201/YN/CHN/2014 | N/A | D3a | this study |
| **2014** | Yunnan | A46/YN/CHN/2014 | N/A | D3a | this study |
| **2014** | Yunnan | J044/YN/CHN/2014 | KY211729 | D3a | this study |
| **2014** | Yunnan | J10/YN/CHN/2014 | N/A | D3a | this study |
| **2014** | Yunnan | J112/YN/CHN/2014 | N/A | D3a | this study |
| **2014** | Yunnan | J119/YN/CHN/2014 | N/A | D3a | this study |
| **2014** | Yunnan | J127/YN/CHN/2014 | N/A | D3a | this study |
| **2014** | Yunnan | J142/YN/CHN/2014 | N/A | D3a | this study |
| **2014** | Yunnan | J15/YN/CHN/2014 | N/A | D3a | this study |
| **2014** | Yunnan | J150/YN/CHN/2014 | N/A | D3a | this study |
| **2014** | Yunnan | J151/YN/CHN/2014 | N/A | D3a | this study |
| **2014** | Yunnan | J16/YN/CHN/2014 | N/A | D3a | this study |
| **2014** | Yunnan | J161/YN/CHN/2014 | N/A | D3a | this study |
| **2014** | Yunnan | J166/YN/CHN/2014 | N/A | D3a | this study |

| | | | | | |
|---|---|---|---|---|---|
| **2014** | Yunnan | J17/YN/CHN/2014 | N/A | D3a | this study |
| **2014** | Yunnan | J178/YN/CHN/2014 | N/A | D3a | this study |
| **2014** | Yunnan | J18/YN/CHN/2014 | N/A | D3a | this study |
| **2014** | Yunnan | J202/YN/CHN/2014 | N/A | D3a | this study |
| **2014** | Yunnan | J22/YN/CHN/2014 | N/A | D3a | this study |
| **2014** | Yunnan | J227/YN/CHN/2014 | N/A | D3a | this study |
| **2014** | Yunnan | J229/YN/CHN/2014 | N/A | D3a | this study |
| **2014** | Yunnan | J23/YN/CHN/2014 | N/A | D3a | this study |
| **2014** | Yunnan | J233/YN/CHN/2014 | KY211730 | D3a | this study |
| **2014** | Yunnan | J252/YN/CHN/2014 | N/A | D3a | this study |
| **2014** | Yunnan | J27/YN/CHN/2014 | N/A | D3a | this study |
| **2014** | Yunnan | J33/YN/CHN/2014 | N/A | D3a | this study |
| **2014** | Yunnan | J37/YN/CHN/2014 | N/A | D3a | this study |
| **2014** | Yunnan | J47/YN/CHN/2014 | N/A | D3a | this study |
| **2014** | Yunnan | J49/YN/CHN/2014 | N/A | D3a | this study |
| **2014** | Yunnan | J5/YN/CHN/2014 | N/A | D3a | this study |
| **2014** | Yunnan | J50/YN/CHN/2014 | N/A | D3a | this study |
| **2014** | Yunnan | J54/YN/CHN/2014 | N/A | D3a | this study |
| **2014** | Yunnan | J58/YN/CHN/2014 | N/A | D3a | this study |
| **2014** | Yunnan | J59/YN/CHN/2014 | N/A | D3a | this study |
| **2014** | Yunnan | J6/YN/CHN/2014 | N/A | D3a | this study |
| **2014** | Yunnan | J60/YN/CHN/2014 | N/A | D3a | this study |
| **2014** | Yunnan | J64/YN/CHN/2014 | KY211732 | D3a | this study |
| **2014** | Yunnan | J74/YN/CHN/2014 | N/A | D3a | this study |
| **2014** | Yunnan | J75/YN/CHN/2014 | N/A | D3a | this study |
| **2014** | Yunnan | J76/YN/CHN/2014 | N/A | D3a | this study |
| **2014** | Yunnan | J80/YN/CHN/2014 | N/A | D3a | this study |
| **2014** | Yunnan | J84/YN/CHN/2014 | N/A | D3a | this study |
| **2014** | Yunnan | J86/YN/CHN/2014 | N/A | D3a | this study |
| **2014** | Yunnan | J87/YN/CHN/2014 | N/A | D3a | this study |
| **2014** | Yunnan | L22/YN/CHN/2014 | KY211733 | D3a | this study |
| **2014** | Yunnan | L45/YN/CHN/2014 | N/A | D3a | this study |
| **2014** | Yunnan | L46/YN/CHN/2014 | N/A | D3a | this study |
| **2014** | Yunnan | L48/YN/CHN/2014 | N/A | D3a | this study |
| **2014** | Yunnan | M27/YN/CHN/2014 | KY211734 | D3a | this study |
| **2014** | Yunnan | M29/YN/CHN/2014 | N/A | D3a | this study |
| **2014** | Yunnan | M30/YN/CHN/2014 | N/A | D3a | this study |
| **2014** | Yunnan | M40/YN/CHN/2014 | N/A | D3a | this study |
| **2014** | Yunnan | N016/YN/CHN/2014 | N/A | D3a | this study |
| **2014** | Yunnan | R109/YN/CHN/2014 | KY211739 | D3a | this study |
| **2014** | Yunnan | R17/YN/CHN/2014 | KY211740 | D3a | this study |
| **2014** | Yunnan | R23/YN/CHN/2014 | N/A | D3a | this study |
| **2015** | Chongqing | 15-22/CQ/CHN/2015 | N/A | D3a | this study |
| **2015** | Chongqing | 15-41/CQ/CHN/2015 | KY424372 | D3a | this study |

| | | | | | |
|---|---|---|---|---|---|
| **2015** | Chongqing | 15-9/CQ/CHN/2015 | KY424367 | D3a | this study |
| **2015** | Gansu | 396/GS/CHN/2015 | KY211703 | D3a | this study |
| **2015** | Gansu | 397/GS/CHN/2015 | N/A | D3a | this study |
| **2015** | Gansu | 400/GS/CHN/2015 | N/A | D3a | this study |
| **2015** | Gansu | 408/GS/CHN/2015 | N/A | D3a | this study |
| **2015** | Gansu | 438/GS/CHN/2015 | KY211704 | D3a | this study |
| **2015** | Gansu | 443/GS/CHN/2015 | KY211705 | D3a | this study |
| **2015** | Gansu | 471/GS/CHN/2015 | N/A | D3a | this study |
| **2015** | Gansu | 493/GS/CHN/2015 | N/A | D3a | this study |
| **2015** | Gansu | 495/GS/CHN/2015 | N/A | D3a | this study |
| **2015** | Gansu | 541/GS/CHN/2015 | KY211709 | D3a | this study |
| **2015** | Gansu | 657/GS/CHN/2015 | KY211712 | D3a | this study |
| **2015** | Gansu | W201510/GS/CHN/2015 | N/A | D3a | this study |
| **2015** | Guangdong | 15-73/GD/CHN/2015 | N/A | D3a | this study |
| **2015** | Guangdong | 15-74/GD/CHN/2015 | N/A | D3a | this study |
| **2015** | Guangdong | 15-75/GD/CHN/2015 | N/A | D3a | this study |
| **2015** | Guangdong | 15-76/GD/CHN/2015 | N/A | D3a | this study |
| **2015** | Guangdong | 15-77/GD/CHN/2015 | N/A | D3a | this study |
| **2015** | Guangdong | 15-78/GD/CHN/2015 | N/A | D3a | this study |
| **2015** | Guangdong | 15-79/GD/CHN/2015 | N/A | D3a | this study |
| **2015** | Guangdong | 15-80/GD/CHN/2015 | N/A | D3a | this study |
| **2015** | Guangdong | 15-81/GD/CHN/2015 | N/A | D3a | this study |
| **2015** | Guangdong | 15-88/GD/CHN/2015 | N/A | D3a | this study |
| **2015** | Guangdong | 15-89/GD/CHN/2015 | N/A | D3a | this study |
| **2015** | Guangdong | 15-92/GD/CHN/2015 | N/A | D3a | this study |
| **2015** | Guangdong | 15-95/GD/CHN/2015 | N/A | D3a | this study |
| **2015** | Guangdong | 15-97/GD/CHN/2015 | N/A | D3a | this study |
| **2015** | Hebei | 1554180/HeB/CHN/2015 | KY211694 | D3a | this study |
| **2015** | Hebei | 1554182/HeB/CHN/2015 | KY211695 | D3a | this study |
| **2015** | Hebei | 1554439/HeB/CHN/2015 | KY211696 | D3a | this study |
| **2015** | Hebei | 1554455/HeB/CHN/2015 | KY211697 | D3a | this study |
| **2015** | Hebei | 1554460/HeB/CHN/2015 | KY211698 | D3a | this study |
| **2015** | Hebei | 1554486/HeB/CHN/2015 | KY211699 | D3a | this study |
| **2015** | Hebei | 1554489/HeB/CHN/2015 | KY211700 | D3a | this study |
| **2015** | Hebei | 54412/HeB/CHN/2015 | N/A | D3a | this study |
| **2015** | Hebei | 54417/HeB/CHN/2015 | N/A | D3a | this study |
| **2015** | Hebei | 54419/HeB/CHN/2015 | N/A | D3a | this study |
| **2015** | Hebei | 54423/HeB/CHN/2015 | N/A | D3a | this study |
| **2015** | Hebei | 54424/HeB/CHN/2015 | N/A | D3a | this study |
| **2015** | Hebei | 54425/HeB/CHN/2015 | N/A | D3a | this study |
| **2015** | Hebei | 54428/HeB/CHN/2015 | N/A | D3a | this study |
| **2015** | Hebei | 54433/HeB/CHN/2015 | N/A | D3a | this study |
| **2015** | Hebei | 54442/HeB/CHN/2015 | N/A | D3a | this study |
| **2015** | Hebei | 54446/HeB/CHN/2015 | N/A | D3a | this study |

| | | | | | |
|---|---|---|---|---|---|
| **2015** | Hebei | 54449/HeB/CHN/2015 | N/A | D3a | this study |
| **2015** | Hebei | 54458/HeB/CHN/2015 | N/A | D3a | this study |
| **2015** | Hebei | 54459/HeB/CHN/2015 | N/A | D3a | this study |
| **2015** | Hebei | 54471/HeB/CHN/2015 | N/A | D3a | this study |
| **2015** | Hebei | 54492/HeB/CHN/2015 | N/A | D3a | this study |
| **2015** | Hebei | 54495/HeB/CHN/2015 | N/A | D3a | this study |
| **2015** | Henan | AYBG15019/HN/CHN/2015 | KU708570 | D3a | GenBank |
| **2015** | Henan | AYLA15007/HN/CHN/2015 | KU708593 | D3a | GenBank |
| **2015** | Henan | AYLA15024/HN/CHN/2015 | KU708594 | D3a | GenBank |
| **2015** | Henan | AYLA15030/HN/CHN/2015 | KU708595 | D3a | GenBank |
| **2015** | Henan | AYLA15035/HN/CHN/2015 | KU708596 | D3a | GenBank |
| **2015** | Henan | AYLA15046/HN/CHN/2015 | KU708597 | D3a | GenBank |
| **2015** | Henan | AYLA15060/HN/CHN/2015 | KU708598 | D3a | GenBank |
| **2015** | Henan | AYLA15151/HN/CHN/2015 | KU708602 | D3a | GenBank |
| **2015** | Henan | AYLA15195/HN/CHN/2015 | KU708604 | D3a | GenBank |
| **2015** | Henan | AYLA15205/HN/CHN/2015 | KU708605 | D3a | GenBank |
| **2015** | Henan | AYLA15218/HN/CHN/2015 | KU708606 | D3a | GenBank |
| **2015** | Henan | AYLA15237/HN/CHN/2015 | KU708607 | D3a | GenBank |
| **2015** | Henan | AYLA15254/HN/CHN/2015 | KU708608 | D3a | GenBank |
| **2015** | Henan | AYLA15270/HN/CHN/2015 | KU708609 | D3a | GenBank |
| **2015** | Henan | AYLA15400/HN/CHN/2015 | KU708615 | D3a | GenBank |
| **2015** | Henan | AYNH15020/HN/CHN/2015 | KU708619 | D3a | GenBank |
| **2015** | Hunan | 15-23/HuN/CHN/2015 | N/A | D3a | this study |
| **2015** | Hunan | 15-5/HuN/CHN/2015 | N/A | D3a | this study |
| **2015** | Hunan | 15-50/HuN/CHN/2015 | KY424369 | D3a | this study |
| **2015** | Hunan | 15-63/HuN/CHN/2015 | KY424368 | D3a | this study |
| **2015** | Jiangxi | 15-16/JX/CHN/2015 | N/A | D3a | this study |
| **2015** | Jiangxi | 15-17/JX/CHN/2015 | N/A | D3a | this study |
| **2015** | Jiangxi | 15-30/JX/CHN/2015 | N/A | D3a | this study |
| **2015** | Jiangxi | 15-43/JX/CHN/2015 | KY424371 | D3a | this study |
| **2015** | Jiangxi | 15-9/JX/CHN/2015 | KY424366 | D3a | this study |
| **2015** | Jiangxi | 15-99/JX/CHN/2015 | KY424364 | D3a | this study |
| **2015** | Liaoning | 18-13/LN/CHN/2015 | KY424363 | D3a | this study |
| **2015** | Liaoning | 18-17/LN/CHN/2015 | N/A | D3a | this study |
| **2015** | Liaoning | 18-23/LN/CHN/2015 | N/A | D3a | this study |
| **2015** | Liaoning | 18-25/LN/CHN/2015 | N/A | D3a | this study |
| **2015** | Liaoning | 18-26/LN/CHN/2015 | N/A | D3a | this study |
| **2015** | Liaoning | 18-29/LN/CHN/2015 | N/A | D3a | this study |
| **2015** | Liaoning | 18-31/LN/CHN/2015 | KY424362 | D3a | this study |
| **2015** | Liaoning | 18-46/LN/CHN/2015 | KY424361 | D3a | this study |
| **2015** | Liaoning | 18-50/LN/CHN/2015 | N/A | D3a | this study |
| **2015** | Liaoning | 18-55/LN/CHN/2015 | N/A | D3a | this study |
| **2015** | Liaoning | 18-60/LN/CHN/2015 | N/A | D3a | this study |
| **2015** | Liaoning | 18-62/LN/CHN/2015 | KY424360 | D3a | this study |

| | | | | | |
|---|---|---|---|---|---|
| **2015** | Liaoning | 18-65/LN/CHN/2015 | N/A | D3a | this study |
| **2015** | Liaoning | 18-68/LN/CHN/2015 | N/A | D3a | this study |
| **2015** | Liaoning | 18-69/LN/CHN/2015 | N/A | D3a | this study |
| **2015** | Liaoning | 18-70/LN/CHN/2015 | N/A | D3a | this study |
| **2015** | Liaoning | 18-9/LN/CHN/2015 | N/A | D3a | this study |
| **2015** | Liaoning | PT-11/LN/CHN/2015 | N/A | D3a | this study |
| **2015** | Liaoning | PT-15/LN/CHN/2015 | N/A | D3a | this study |
| **2015** | Liaoning | PT-6/LN/CHN/2015 | KY424356 | D3a | this study |
| **2015** | Shandong | BZ034/SD/CHN/2015 | KY211717 | D3a | this study |
| **2015** | Shandong | BZ083/SD/CHN/2015 | KY211718 | D3a | this study |
| **2015** | Shandong | BZ103/SD/CHN/2015 | N/A | D3a | this study |
| **2015** | Shandong | BZ194/SD/CHN/2015 | N/A | D3a | this study |
| **2015** | Shandong | DY033/SD/CHN/2015 | KY211721 | D3a | this study |
| **2015** | Shandong | DY081/SD/CHN/2015 | KY211722 | D3a | this study |
| **2015** | Shandong | DY088/SD/CHN/2015 | N/A | D3a | this study |
| **2015** | Shandong | DZ406/SD/CHN/2015 | N/A | D3a | this study |
| **2015** | Shandong | JA277/SD/CHN/2015 | N/A | D3a | this study |
| **2015** | Shandong | JA279/SD/CHN/2015 | N/A | D3a | this study |
| **2015** | Shandong | WF415/SD/CHN/2015 | KY211741 | D3a | this study |
| **2015** | Shandong | YT03/SD/CHN/2015 | N/A | D3a | this study |
| **2015** | Shandong | YT07/SD/CHN/2015 | N/A | D3a | this study |
| **2015** | Shandong | YT08/SD/CHN/2015 | N/A | D3a | this study |
| **2015** | Shandong | YT12/SD/CHN/2015 | N/A | D3a | this study |
| **2015** | Shandong | YT17/SD/CHN/2015 | N/A | D3a | this study |
| **2015** | Shandong | ZZ145/SD/CHN/2015 | N/A | D3a | this study |
| **2015** | Shanghai | SHAPHC5696/SH/CHN/15 | KU736939 | D3a | GenBank |
| **2015** | Shanghai | SHAPHC5793/SH/CHN/15 | KU736937 | D3a | GenBank |
| **2015** | Shanghai | SHAPHC5881/SH/CHN/15 | KU736935 | D3a | GenBank |
| **2015** | Shanghai | SHAPHC5883/SH/CHN/15 | KU736938 | D3a | GenBank |
| **2015** | Shanghai | SHAPHC5949/SH/CHN/15 | KU736934 | D3a | GenBank |
| **2015** | Shanghai | SHAPHC5981/SH/CHN/15 | KU736940 | D3a | GenBank |
| **2015** | Shanghai | SHAPHC6029/SH/CHN/15 | KU736933 | D3a | GenBank |
| **2015** | Shanghai | SHAPHC6049/SH/CHN/15 | KU736941 | D3a | GenBank |
| **2015** | Shanghai | SHAPHC6084/SH/CHN/15 | KU736932 | D3a | GenBank |
| **2015** | Shanghai | SHAPHC6115/SH/CHN/15 | KU736942 | D3a | GenBank |
| **2015** | Shanghai | SHAPHC6141/SH/CHN/15 | KU736931 | D3a | GenBank |
| **2015** | Shanghai | SHAPHC6188/SH/CHN/15 | KU736936 | D3a | GenBank |
| **2015** | Sichuan | 15-25/SC/CHN/2015 | N/A | D3a | this study |
| **2015** | Sichuan | 15-28/SC/CHN/2015 | N/A | D3a | this study |
| **2015** | Sichuan | 15-29/SC/CHN/2015 | N/A | D3a | this study |
| **2015** | Sichuan | 15-49/SC/CHN/2015 | KY424370 | D3a | this study |
| **2015** | Tianjin | 15-16/TJ/CHN/2015 | N/A | D3a | this study |
| **2015** | Tianjin | 15-34/TJ/CHN/2015 | N/A | D3a | this study |
| **2015** | Tianjin | 15-9/TJ/CHN/2015 | N/A | D3a | this study |

| | | | | | |
|---|---|---|---|---|---|
| **2015** | Xinjiang | 15-91/XJ/CHN/2015 | KY424365 | D3a | this study |
| **2015** | Yunnan | A1/YN/CHN/2015 | KY211714 | D3a | this study |
| **2015** | Yunnan | A113/YN/CHN/2015 | N/A | D3a | this study |
| **2015** | Yunnan | A120/YN/CHN/2015 | KY424357 | D3a | this study |
| **2015** | Yunnan | A13/YN/CHN/2015 | N/A | D3a | this study |
| **2015** | Yunnan | A2/YN/CHN/2015 | N/A | D3a | this study |
| **2015** | Yunnan | A5/YN/CHN/2015 | N/A | D3a | this study |
| **2015** | Yunnan | A67/YN/CHN/2015 | KY211716 | D3a | this study |
| **2015** | Yunnan | A72/YN/CHN/2015 | N/A | D3a | this study |
| **2015** | Yunnan | A97/YN/CHN/2015 | N/A | D3a | this study |
| **2015** | Yunnan | C1/YN/CHN/2015 | N/A | D3a | this study |
| **2015** | Yunnan | C105/YN/CHN/2015 | N/A | D3a | this study |
| **2015** | Yunnan | C106/YN/CHN/2015 | N/A | D3a | this study |
| **2015** | Yunnan | C108/YN/CHN/2015 | N/A | D3a | this study |
| **2015** | Yunnan | C109/YN/CHN/2015 | N/A | D3a | this study |
| **2015** | Yunnan | C116/YN/CHN/2015 | N/A | D3a | this study |
| **2015** | Yunnan | C120/YN/CHN/2015 | N/A | D3a | this study |
| **2015** | Yunnan | C122/YN/CHN/2015 | N/A | D3a | this study |
| **2015** | Yunnan | C125/YN/CHN/2015 | N/A | D3a | this study |
| **2015** | Yunnan | C126/YN/CHN/2015 | N/A | D3a | this study |
| **2015** | Yunnan | C134/YN/CHN/2015 | N/A | D3a | this study |
| **2015** | Yunnan | C135/YN/CHN/2015 | N/A | D3a | this study |
| **2015** | Yunnan | C136/YN/CHN/2015 | N/A | D3a | this study |
| **2015** | Yunnan | C139/YN/CHN/2015 | N/A | D3a | this study |
| **2015** | Yunnan | C140/YN/CHN/2015 | N/A | D3a | this study |
| **2015** | Yunnan | C141/YN/CHN/2015 | KY211719 | D3a | this study |
| **2015** | Yunnan | C143/YN/CHN/2015 | N/A | D3a | this study |
| **2015** | Yunnan | C144/YN/CHN/2015 | N/A | D3a | this study |
| **2015** | Yunnan | C145/YN/CHN/2015 | N/A | D3a | this study |
| **2015** | Yunnan | C147/YN/CHN/2015 | N/A | D3a | this study |
| **2015** | Yunnan | C148/YN/CHN/2015 | N/A | D3a | this study |
| **2015** | Yunnan | C150/YN/CHN/2015 | N/A | D3a | this study |
| **2015** | Yunnan | C157/YN/CHN/2015 | N/A | D3a | this study |
| **2015** | Yunnan | C159/YN/CHN/2015 | N/A | D3a | this study |
| **2015** | Yunnan | C160/YN/CHN/2015 | N/A | D3a | this study |
| **2015** | Yunnan | C162/YN/CHN/2015 | N/A | D3a | this study |
| **2015** | Yunnan | C171/YN/CHN/2015 | N/A | D3a | this study |
| **2015** | Yunnan | C174/YN/CHN/2015 | N/A | D3a | this study |
| **2015** | Yunnan | C177/YN/CHN/2015 | N/A | D3a | this study |
| **2015** | Yunnan | C2/YN/CHN/2015 | N/A | D3a | this study |
| **2015** | Yunnan | C26/YN/CHN/2015 | N/A | D3a | this study |
| **2015** | Yunnan | C3/YN/CHN/2015 | N/A | D3a | this study |
| **2015** | Yunnan | C42/YN/CHN/2015 | N/A | D3a | this study |
| **2015** | Yunnan | C45/YN/CHN/2015 | N/A | D3a | this study |

| | | | | | |
|---|---|---|---|---|---|
| **2015** | Yunnan | C46/YN/CHN/2015 | N/A | D3a | this study |
| **2015** | Yunnan | C48/YN/CHN/2015 | N/A | D3a | this study |
| **2015** | Yunnan | C53/YN/CHN/2015 | N/A | D3a | this study |
| **2015** | Yunnan | C6/YN/CHN/2015 | N/A | D3a | this study |
| **2015** | Yunnan | C61/YN/CHN/2015 | N/A | D3a | this study |
| **2015** | Yunnan | C63/YN/CHN/2015 | N/A | D3a | this study |
| **2015** | Yunnan | C67/YN/CHN/2015 | N/A | D3a | this study |
| **2015** | Yunnan | C68/YN/CHN/2015 | N/A | D3a | this study |
| **2015** | Yunnan | C69/YN/CHN/2015 | N/A | D3a | this study |
| **2015** | Yunnan | C70/YN/CHN/2015 | N/A | D3a | this study |
| **2015** | Yunnan | C71/YN/CHN/2015 | N/A | D3a | this study |
| **2015** | Yunnan | C76/YN/CHN/2015 | N/A | D3a | this study |
| **2015** | Yunnan | C77/YN/CHN/2015 | N/A | D3a | this study |
| **2015** | Yunnan | C89/YN/CHN/2015 | N/A | D3a | this study |
| **2015** | Yunnan | C9/YN/CHN/2015 | N/A | D3a | this study |
| **2015** | Yunnan | C92/YN/CHN/2015 | KY211720 | D3a | this study |
| **2015** | Yunnan | C93/YN/CHN/2015 | N/A | D3a | this study |
| **2015** | Yunnan | C95/YN/CHN/2015 | N/A | D3a | this study |
| **2015** | Yunnan | C97/YN/CHN/2015 | N/A | D3a | this study |
| **2015** | Yunnan | J24/YN/CHN/2015 | KY211731 | D3a | this study |
| **2015** | Yunnan | J28/YN/CHN/2015 | N/A | D3a | this study |
| **2015** | Yunnan | J36/YN/CHN/2015 | N/A | D3a | this study |
| **2015** | Yunnan | J37/YN/CHN/2015 | N/A | D3a | this study |
| **2015** | Yunnan | N15/YN/CHN/2015 | N/A | D3a | this study |
| **2015** | Yunnan | N17/YN/CHN/2015 | KY211735 | D3a | this study |
| **2015** | Yunnan | N18/YN/CHN/2015 | N/A | D3a | this study |
| **2015** | Yunnan | N21/YN/CHN/2015 | N/A | D3a | this study |
| **2015** | Yunnan | N28/YN/CHN/2015 | N/A | D3a | this study |
| **2015** | Yunnan | N30/YN/CHN/2015 | N/A | D3a | this study |
| **2015** | Yunnan | N4/YN/CHN/2015 | KY211738 | D3b | this study |
